# Supplementary material for: Comparative efficacy and acceptability of antiepileptic drugs for classical trigeminal neuralgia: a Bayesian network meta-analysis protocol
Source: BMJ Open. 2018 Jan 21;8(1):e017392. doi: 10.1136/bmjopen-2017-017392 (PMC5780694; doi:10.1136/bmjopen-2017-017392)
Supplement: Supplementary file 2 [file bmjopen-2017-017392supp002.pdf]

PubMed

Patient

#1 "Trigeminal Neuralgia"[Mesh]

#2 Trigeminal Neuralgia*[Title/Abstract] OR Trifacial Neuralgia*[Title/Abstract] OR Fothergill Disease[Title/Abstract] OR Tic Douloureux[Title/Abstract] OR Epileptiform Neuralgia*[Title/Abstract] OR trigeminus neuralgia[Title/Abstract] OR prosopalgia[Title/Abstract] OR prosoponeuralgia[Title/Abstract] OR trigeminal nerve neuralgia[Title/Abstract] OR trigeminal nerve neuropathy[Title/Abstract] OR trigeminal neuropathy[Title/Abstract] OR trigeminus nerve neuralgia[Title/Abstract] OR trigeminus nerve neuropathy[Title/Abstract]

#3 #1 OR #2

RCT

#4 "Clinical Trials, Phase II as Topic"[Mesh] OR "Clinical Trials, Phase III as Topic"[Mesh] OR "Clinical Trials, Phase IV as Topic"[Mesh] OR "Controlled Clinical Trials as Topic"[Mesh] OR "Randomized Controlled Trials as Topic"[Mesh] OR "Intention to Treat Analysis"[Mesh] OR "Pragmatic Clinical Trials as Topic"[Mesh] OR "Clinical Trials, Phase II"[Publication Type] OR "Clinical Trials, Phase III"[Publication Type] OR "Clinical Trials, Phase IV"[Publication Type] OR "Controlled Clinical Trials"[Publication Type] OR "Randomized Controlled Trials"[Publication Type] OR "Pragmatic Clinical Trials as Topic"[Publication Type] OR "Single-Blind Method"[Mesh] OR "Double-Blind Method"[Mesh]

#5 random*[Title/Abstract] OR blind*[Title/Abstract] OR singleblind*[Title/Abstract] OR doubleblind*[Title/Abstract] OR trebleblind*[Title/Abstract] OR tripleblind*[Title/Abstract]

#6 #4 OR #5

#7 #3 AND #6

EMBASE.com

Patient

#1 'trigeminus neuralgia'/exp

#2 'trigeminal neuralgias':ab,ti OR 'trifacial neuralgias':ab,ti OR 'trigeminal neuralgia':ab,ti OR 'trifacial neuralgia':ab,ti OR 'fothergill disease':ab,ti OR 'tic douloureux':ab,ti OR 'epileptiform neuralgia':ab,ti OR 'epileptiform neuralgias':ab,ti OR 'trigeminus neuralgia':ab,ti OR prosopalgia:ab,ti OR prosoponeuralgia:ab,ti OR 'trigeminal nerve neuralgia':ab,ti OR 'trigeminal nerve neuropathy':ab,ti OR 'rigeminal neuropathy':ab,ti OR 'trigeminus nerve neuralgia':ab,ti OR 'trigeminus nerve neuropathy':ab,ti

#3 #1 OR #2

RCT

#4 'multicenter study (topic)'/exp OR 'phase 2 clinical trial (topic)'/exp OR 'phase 3 clinical trial

(topic)'/exp OR 'phase 4 clinical trial (topic)'/exp OR 'controlled clinical trial (topic)'/exp OR 'randomized controlled trial (topic)'/exp OR 'single blind procedure'/exp OR 'double blind procedure'/exp

#5 random*:ab,ti OR blind*:ab,ti OR singleblind*:ab,ti OR doubleblind*:ab,ti OR trebleblind*:ab,ti OR tripleblind*:ab,ti

#6 #4 OR #5

#7 #3 AND #6

Cochrane Library

#1 MeSH descriptor: [Trigeminal Neuralgia] explode all trees

#2 Trigeminal Neuralgia*:ti,ab,kw or Trifacial Neuralgia*:ti,ab,kw or Fothergill Disease:ti,ab,kw or Tic Douloureux:ti,ab,kw or Epileptiform Neuralgia*:ti,ab,kw or trigeminus neuralgia:ti,ab,kw or prosopalgia:ti,ab,kw or prosoponeuralgia:ti,ab,kw or trigeminal nerve neuralgia:ti,ab,kw or trigeminal nerve neuropathy:ti,ab,kw or trigeminal neuropathy:ti,ab,kw or trigeminus nerve neuralgia:ti,ab,kw or trigeminus nerve neuropathy:ti,ab,kw

#3 #1 or #2
