# Supplementary material for: Comparative efficacy and acceptability of antiepileptic drugs for classical trigeminal neuralgia: a Bayesian network meta-analysis protocol
Source: BMJ Open. 2018 Jan 21;8(1):e017392. doi: 10.1136/bmjopen-2017-017392 (PMC5780694; doi:10.1136/bmjopen-2017-017392)
Supplement: Author's manuscript [file bmjopen-2017-017392.draft_revisions.pdf]

BMJ Open is committed to open peer review. As part of this commitment we make the peer review history of every article we publish publicly available.

When an article is published we post the peer reviewers' comments and the authors' responses online. We also post the versions of the paper that were used during peer review. These are the versions that the peer review comments apply to.

The versions of the paper that follow are the versions that were submitted during the peer review process. They are not the versions of record or the final published versions. They should not be cited or distributed as the published version of this manuscript.

BMJ Open is an open access journal and the full, final, typeset and author-corrected version of record of the manuscript is available on our site with no access controls, subscription charges or pay-per-view fees (http://bmjopen.bmj.com).

If you have any questions on BMJ Open's open peer review process please email editorial.bmjopen@bmj.com

BMJ Open

Comparative Efficacy and Acceptability of Antiepileptic Drugs for Classical Trigeminal Neuralgia: A Bayesian Network Meta-Analysis Protocol

Journal:	*BMJ Open*
Manuscript ID	bmjopen-2017-017392
Article Type:	Protocol
Date Submitted by the Author:	20-Apr-2017
Complete List of Authors:	Qin, Zongshi; Guang'anmen Hospital, China Academy of Chinese Medical Sciences, Department of Acupuncture and Neurology<br>Xie, Shang; Peking University School and Hospital of Stomatology, Department of Oral and Maxillofacial Surgery<br>Mao, Zhi; Chinese People's Liberation Army General Hospital, Department of Critical Care Medicine<br>Liu, Yan; China Academy of Chinese Medical Sciences, Data Centre of Traditional Chinese Medicine<br>WU, Jiani; Guang'anmen Hospital, China Academy of Chinese Medical Sciences, Department of Acupuncture and Neurology<br>Furukawa, Toshi; Kyoto University, Graduate School of Medicine and School of Public Health<br>Kwong, Joey; Taipei Medical University, Cochrane Taiwan; National Center for Child Health and Development, Department of Health Policy & Department of Clinical Epidemiology<br>Tian, Jinhui; Evidence based medicine center,<br>Liu, Zhishun; Guang'anmen Hospital, China Academy of Chinese Medical Sciences, Department of Acupuncture and Neurology
**Primary Subject Heading**:	Evidence based practice
Secondary Subject Heading:	Neurology
Keywords:	antiepileptic drugs, trigeminal neuralgia, network meta-analysis, systematic review, protocol

SCHOLARONE™ Manuscripts

Comparative Efficacy and Acceptability of Antiepileptic Drugs for Classical Trigeminal Neuralgia: A Bayesian Network Meta-Analysis Protocol

Zongshi Qin,[1] Shang Xie[2], Zhi Mao[3], Yan Liu[4], Jiani Wu[1], Toshi A Furukawa[5], Joey S.W. Kwong[6,7], Jinhui Tian[8], Zhishun Liu[1]

[1] Department of Acupuncture and Neurology, Guang'anmen Hospital, China Academy of Chinese Medical Sciences, Beijing, China

[2] Department of Oral and Maxillofacial Surgery, Peking University School and Hospital of Stomatology, Beijing, China

[3] Department of Critical Care Medicine, Chinese People's Liberation Army General Hospital, Beijing, China

[4] Data Centre of Traditional Chinese Medicine, China Academy of Chinese Medical Sciences, Beijing, China

[5] Department of Health Promotion and Human Behavior, Kyoto University Graduate School of Medicine/School of Public Health, Kyoto, Japan

[6] Cochrane Taiwan, Taipei Medical University, Taipei, Taiwan

[7] Department of Health Policy & Department of Clinical Epidemiology, National Center for Child Health and Development, Tokyo, Japan

[8] Evidence-based Medicine Center, Lanzhou University, Lanzhou, China

ZQ, SX and ZM contributed equally to this work.

Correspondence to: Jinhui Tian, email: tianjh@lzu.edu.cn; Zhishun Liu, email: liuzhishun@aliyun.com

Keywords: antiepileptic drugs; trigeminal neuralgia; network meta-analysis; systematic review; protocol

For peer review only - http://bmjopen.bmj.com/site/about/guidelines.xhtml

ABSTRACT

Introduction As the most common neuralgia, trigeminal neuralgia (TN) affects 4 to 28.9/100,000 people worldwide, and antiepileptic drugs such as carbamazepine and oxcarbazepine are the first-line treatment options. However, the efficacy and safety of other antiepileptic drugs remain unclear due to insufficient direct comparisons.

Objective To compare the efficacy and acceptability of all currently available antiepileptic agents for the treatment of patients with classical TN.

Methods We will search the PubMed, EMBASE, Cochrane Library, and Web of Science databases for unpublished or undergoing research listed in registry platforms. We will include all randomized controlled trials comparing two different antiepileptic drugs or one antiepileptic drug with placebo in patients with classic TN. The primary outcomes will be the proportion of responders and the number of subjects who drop out during the treatment. The secondary outcomes include the two primary outcomes but set in the follow-up period, changes in the self-reporting assessment scale for neuralgia, and quality of life assessment. In terms of network meta-analysis, we will fit our model in a Bayesian framework using WinBUGS (Medical Research Council's Biostatistics Unit, Cambridge, UK). To confirm the results, we will also conduct analyses using STATA (Version 13.0; Stata Corporation, College Station, Texas, USA) and compare the differences between the two platforms.

Ethics and dissemination This protocol will not disseminate any private patient data.

Protocol registration for this systematic review (registration number): PROSPERO (CRD: 42016048640).

Strengths and limitations of this study

• To the best of the authors' knowledge, this study will be the first network meta-analysis that assess the comparative efficacy and acceptability of all the available antiepileptic drugs for the classical trigeminal neuralgia.

• This study will be performed by Bayesian framework, which enables us to estimate the probability for each intervention to be the best for each outcome.

• Owing to the language barrier, the amount of included trials might be potentially limited.

For peer review only - http://bmjopen.bmj.com/site/about/guidelines.xhtml

Introduction

Classical trigeminal neuralgia (TN), a chronic pain disorder described as one of the most severe pains one can suffer, is characterized by paroxysms of unilateral, electric shock-like, and severe pain along the trigeminal nerve divisions.[1,2] It affects lifestyle because it can be triggered by common activities such as eating, talking, shaving and brushing teeth. The wind, chewing and talking also aggravate the condition in many patients.[2] As the most common neuralgia, it is estimated that approximately 4 to 28.9 per 100,000 people worldwide suffer from TN, and the number affected tends to be higher among women at all ages and even increases with age.[3,4]

At present, the cause of TN remains unclear.[5,6] One of the most common hypotheses is that the trigeminal nerve becomes compressed at the root entry zone by cerebral vessels.[7] Owing to the contradictory etiology and poorly understood pathophysiological mechanisms underlying TN, a variety of therapeutic and surgical approaches have been developed to alleviate the associated pain and improve the quality of life in patients with classical TN.[8-10] Although many patients have obtained excellent outco mes from surgery, many others do not experience any pain relief.[11,12] Furthermore, the currently available surgical procedures are associated with various complications, particularly sensory loss in the trigeminal nerve territory, anesthesia dolorosa and, rarely, ipsilateral hearing loss, depending on the technique.[13-15]

As such, pharmacological measures to improve clinical outcomes are needed. The most commonly used option is antiepileptic drugs, with phenytoin being the first drug used for classical TN with positive effect.[16] Carbamazepine can reduce both the frequency and intensity of painful paroxysms and was first introduced by the US Food and Drug Administration (FDA); however, its efficacy is compromised by poor tolerability.[17,18] Oxcarbazepine, a derivative of carbamazepine, is often used as an initial treatment for classical TN and has more favorable properties than carbamazepine related to its increased efficacy in epilepsy, greater tolerability and decreased potential for drug interactions.[17,18] Lamotrigine has also been reported as an effective add-on therapy,[19] whereas little evidence supports that other antiepileptic drugs such as clonazepam, gabapentin, pregabalin and valproate have a beneficial effect.[20-23]

To date, several systematic reviews have investigated the comparative efficacy and safety of antiepileptic drugs.[21,24-28] However, previous systematic reviews have only considered pair-wise evidence from head-to-head comparisons and have thus failed to assess the comparative efficacy

For peer review only - http://bmjopen.bmj.com/site/about/guidelines.xhtml

and acceptability of all available antiepileptic drugs. Thus, it is difficult to determine the best treatments for relieving pain with minimal adverse effects. In the present study, we will apply network meta-analysis to integrate direct and indirect comparisons,[29,30] which could be used not only to strengthen inferences concerning the efficacy and acceptability of treatments but also to rank the efficacy and acceptability of antiepileptic drugs accordingly.[31]

The objectives of this systematic review and network meta-analysis are 1) to compare all currently available antiepileptic drugs in terms of efficacy and acceptability in classical TN treatment and 2) to determine which drug achieves the best balance between efficacy and adverse effects. The results of this study will augment findings based on current pair-wise meta-analyses and are expected to provide important information to support clinical practice and health policy decisions.

METHODS

This protocol will be conducted in accordance with the reporting guidance provided in the Preferred Reporting Items for Systematic Reviews and Meta-Analysis Protocols (PRISMA-P) statement and Checklist of Items to Include When Reporting a Systematic Review Involving a Network Meta-analysis.[32,33] The protocol is registered in PROSPERO (CRD: 42016048640). This study will not involve any private patient data, ethical approval was waived. (supplemental file 1 represents the PRISMA-P checklist)

Eligibility criteria

Study types

We will include randomized controlled trials (RCTs) comparing one antiepileptic drug with another antiepileptic drug as monotherapy or placebo for the treatment of TN. Quasi-randomized controlled trails allocating participants according to birth date or the consequences of enrollment will be excluded. The minimum duration for RCT inclusion was set at 4 weeks. Trials with more than a two-arm design will be considered only if the available data meet the criteria for an intervention. For trials with a crossover design, data will only be extracted from the first randomization period.

Participant characteristics

For peer review only - http://bmjopen.bmj.com/site/about/guidelines.xhtml

Only trials that enrolled participants with a diagnosis of classical TN according to standardized criteria such as the classification of the International Headache Society (IHS) of International Classification of Headache Disorders will be sought.[1,34] For studies using other extensive criteria for the diagnosis of classical TN, detailed diagnostic criteria must be reported (such as history or characteristics that have been confirmed by CT or MRI).[35] Studies examining symptomatic TN patients will not be included. Participants with comorbid conditions such as anxiety, depression, epilepsy or other medical conditions will also not be eligible for inclusion. No limitations will be imposed for age, sex, or nationality.

Intervention types

We plan to include the following antiepileptic drugs: carbamazepine, lamotrigine, clonazepam, phenytoin, valproate, gabapentin, pregabalin, oxcarbazepine and topiramate. In addition to these antiepileptic drugs, we will also obtain information about interventions of interest from either pair-wise RCTs or placebo-controlled trails, as some RCTs design a placebo-controlled arm as the comparator. Figure 1 illustrates the network plot of all possible direct comparisons between the eligible interventions.

Outcome measures

Studies reporting one of the following will be included.

Primary outcome

The primary objective of this review is to assess the efficacy and acceptability of antiepileptic drugs for classical TN; therefore, the following two outcomes will be used as the primary outcome.

1. The proportion of responders to a self-reporting assessment scale for neuralgia. A responder was defined as a subject who obtained a ≥ 50% pain reduction score from baseline to endpoint (4–12 weeks) or a subject who obtained a pain reducing score of no less than the minimal clinically important difference (MCID). Pain scores will be extracted based on the visual analogue score (VAS), numerical rating score (NRS), or any other validated scale for the assessment of overall TN symptoms when available.[36]

2. The proportion of participants who drop out from a study from baseline to endpoint (4–12

For peer review only - http://bmjopen.bmj.com/site/about/guidelines.xhtml

weeks) due to adverse events, defined as events resulting from any factor during treatment.

Secondary outcomes

1. The proportion of responders with ≥ 50% pain reduction on a self-reporting assessment scale for neuralgia from baseline to endpoint after follow-up.

2. The change in pain symptoms of TN from baseline to endpoint (4–12 weeks) measured based on the VAS, NRS, or any other validated scale for the assessment of overall TN symptoms when available.

3. The change in pain symptoms of TN from baseline to endpoint after follow-up.

4. The quality of life based on measurement with a validated scale, such as the Short Form 36 Health Survey questionnaire (SF-36).[37]

Search strategy

We will identify RCTs through a comprehensive, systematic literature search primarily utilizing the PubMed, EMBASE, Cochrane Library, and Web of Science databases. As publication bias caused by insufficient unpublished data can significantly bias the comparative efficacy results of network meta-analysis and modify rankings, we will also perform searches for unpublished or ongoing trials using System for information on Grey Literature in Europe (SIGLE) as well as other registry platforms, such as Clinicaltrials.gov and the International Clinical Trials Registry Platform. Prior to completing this review, we will perform an additional search of each database and registration platform to guarantee that the most recent studies are included. We will use medical subject headings and text words related to 'trigeminal neuralgia' and 'randomized controlled trial' for the literature search. In addition, the reference lists of previous systematic reviews will be examined to ensure the quantity and accuracy of the included studies. The search strategy will be developed by JT and ZL. (supplemental file 2 represents the search strategies for PubMed, EMBASE and Cochrane Library)

Data collection process

Two authors (SX and ZM) will scan the titles and abstracts of the trials after duplicated records have been excluded using EndNote X7 (Thomson Reuters, New York, NY). The scanning will be

For peer review only - http://bmjopen.bmj.com/site/about/guidelines.xhtml

performed using EndNote, and all trials will be allocated to the following five groups: inclusion group, non-patient group, intervention group, outcome group, and awaiting group. A prior data collection process will be conducted using an electro-table created with Excel software, which has been used in our previous study.[38] The table will consist of four sheets, including general information (author list, publication year, and journal), characteristics of included trials (diagnostic criteria, age range, study drugs, and dose range), the risk of bias assessed using the Cochrane risk of bias tool, and outcome data extraction (number of participants who responded to treatment and the number who dropped out during the treatment). All original data will be submitted as an attachment. A flow chart illustrating this design is presented in Figure 2.

Quality assessment

Two authors (JW and YL) will use the Cochrane risk of bias tool to assess the risk of bias of eligible studies, covering randomization, concealment allocation, blinding and other biases.[39] As inadequate concealment could potentially fail the randomization test, two independent review authors will pay particular attention to the adequacy of random allocation concealment and blinding. The other sources of bias will be assessed while considering sample size calculation method, diagnostic criteria, reporting of withdrawals and follow-up. Two authors (JK and JT) will assess the quality of evidence using Grading of Recommendations Assessment, Development and Evaluation (GRADE) framework, covering study limitations, inconsistency, indirectness, imprecision and publication bias.[40] The methods for rating the quality of direct comparisons are the same to the methods used in traditional meta-analysis, and following steps will be used in the whole assessment procedure: 1) presenting direct and indirect effect estimates; 2) rating the quality of direct and indirect estimates; 3) presenting the results of network meta-analysis; 4) rating the quality of network meta-analysis effect estimates.

Dealing with missing data

To obtain missing data, we will initially contact the senior or corresponding author. If no one responds, we will estimate the missing data as follows. For studies failing to report the number of responding patients after treatment, instead of providing the mean and standard deviation, we will calculate the number of responding patients employing a validated imputation method.[41] In

For peer review only - http://bmjopen.bmj.com/site/about/guidelines.xhtml

addition, we will also estimate missing data from graphs when possible. For trials that cannot be extracted or estimated, the available data will be excluded, and the reason for exclusion will be reported.

Statistical analysis

The method used for data synthesis will be based on mixed treatment meta-analysis. To examine comparisons, we will use STATA (Version 13.0; Stata Corporation, College Station, Texas, USA) to synthesize data and will present the comparison results if the included studies are sufficient for each pair-wise comparison. We will use a random effects model to combine the data, and the outcomes of continuous and binary variables will be presented as standardized mean differences (SMDs) and odds ratios (ORs) with 95% confidential intervals (CIs). For indirect comparisons, a random effects model network meta-analysis will be developed in a Bayesian framework using Markov chain Monte Carlo simulation methods in WinBUGS (Medical Research Council's Biostatistics Unit, Cambridge, UK). This will enable us to estimate the best probability for each intervention for each positive outcome, given the results of the multiple-treatment meta-analysis. At least one network focusing on the response rate for pain relief will be constructed, in which a statistically significant difference defined as the null value will not be included in the 95% CI. The Markov chains will be utilized for 50,000 simultaneous iterations based on the data and the description of the proposed distributions for relevant parameters, and the first 10,000 iterations will be discarded to avoid potential impact on the arbitrary value. For continuous outcomes and binary outcomes, the OR and SMD will be presented with the 95% credible interval (CrI). In this process, the Brooks-Gelman-Rubin method will be used to assess the convergence between direct and indirect variances. According to the theory of Brooks and Gelman, if a potential scale reduction factor (PSRF) is less than 1.2, then an approximate convergence has occurred. The PSRF results will be presented graphically using a Brooks-Gelman-Rubin diagnosis plot, if needed. To describe relationships among different treatments, a network plot will be created to show direct comparisons between arms based on different outcomes. To confirm the results, we will also conduct the same network meta-analysis using the network package of STATA (http://www.mrc-bsu.cam.ac.uk/IW_Stata/), and the outcome will be compared to that produced using WinBUGS. In addition, the effectiveness of each treatment among all available treatments

For peer review only - http://bmjopen.bmj.com/site/about/guidelines.xhtml

For peer review only

will be ranked by calculating the OR in order, and plots of the surfaces under the cumulative ranking curves (SUCRAs) will be generated to rank the various treatments for each outcome.[42] We will also present a cluster rank table to synthesize the efficacy and acceptability of each drug (using two primary outcomes). The table will consist of two triangles: the upper right triangle will illustrate the acceptability, and the lower left triangle will illustrate the efficacy.[31]

Assessment of heterogeneity

Heterogeneity, which plays a pivotal role in both standard meta-analysis and network meta-analysis, refers to the degree of disagreement between study-specific treatment effects and constitutes the basis of inconsistency. To test the heterogeneity of each pair-wise comparison, we will use the I^2 statistic.[43]

Assessment of transitivity and similarity

In addition to the heterogeneity assessment using the I^2 statistic, the assumption of transitivity and similarity based on clinical and methodological characteristics will be assessed. It should be noted that it is difficult to identify these effect modifiers using statistical analysis. We will assume that intervention effects are transitive in this network meta-analysis because we will only focus on antiepileptic drugs, and we will investigate similarity based on clinical characteristics, such as antiepileptic drug dose, period of treatment, and severity of pain symptoms at baseline, as well as according to methodological characteristics such as study quality.[44] All these effect modifiers will be judged and reported before the network meta-analysis is conducted.

Assessment of inconsistency

The evaluation and explanation of inconsistency is another basic objective of network meta-analysis. In this context, inconsistency refers to the degree of difference between direct and indirect comparisons and can be evaluated only when a loop exists in the evidence network. This means that inconsistency assessment using a design-by-treatment interaction model cannot be conducted if the structure of this network is a "star network" (i.e., all interventions have a single mutual comparator, such as a placebo).[45,46] For such cases, we will test inconsistency using a node-splitting model.[47]

To identify inconsistency among the included trials of the network, we will use STATA,

For peer review only - http://bmjopen.bmj.com/site/about/guidelines.xhtml

performing the Z test to compare direct and indirect summary effects in specific loops.[48] If there is no inconsistency between loops or designs, we will use a consistency model to calculate the data. For cases of significant incoherence, we will initially look for data extraction errors in loops that present inconsistency and in comparisons with large heterogeneity.[49] After the data have been scrutinized, we will investigate possible sources of inconsistency within clinical and methodological variables suspected of being potential sources of either heterogeneity or incoherence in each comparison-specific group of trials. If an important inconsistency cannot be explained, we will consider avoiding synthesis of the related network.

Additional analyses

To ensure the quality of this review, studies not reporting blinding will be excluded prior to data synthesis because blinding plays a vital important role in the randomized controlled trial. We will assess heterogeneity quantitatively using the I^2 statistic, and if an I^2 value is greater than 50%, then we will explore the source of heterogeneity. We will initially perform sensitivity analysis by excluding trials rated as having a high risk of bias. Additionally, meta-regression or subgroup analysis will be used to explore possible sources of heterogeneity if the number of included trials is sufficient. For network meta-regression, we will use a random effects network meta-regression model to examine potential factors.

Discussion

To the best of our knowledge, no network meta-analyses comparing the use of antiepileptic drugs for treatment of classical TN have been conducted to date. Previous systematic reviews have compared only a single drug to other types of drug or therapy.[21,24-28] This makes it difficult to obtain a clear understanding of the effectiveness of the various different conservative treatments for this disorder. Network meta-analysis can be used to perform indirect comparisons and allows parameters for direct and indirect comparisons to be synthesized. To ensure the quantity and quality of the potentially included RCTs, we will perform an extensive literature search and predefine rigorous inclusion criteria. Besides, we will assess the quality of evidence with the GRADE framework. Although a ranking of the included interventions will be generated, with the exception of findings, the quality of evidence should also be considered. We hope that the results

For peer review only - http://bmjopen.bmj.com/site/about/guidelines.xhtml

For peer review only

of this review help clinicians make more accurate treatment decisions and promote additional research into conservative treatments for classical TN.

For peer review only

For peer review only - http://bmjopen.bmj.com/site/about/guidelines.xhtml

Amendments

If it is necessary to update this protocol, we will update this protocol in the future. We will submit the original protocol, final protocol and summary of changes as a supplement.

Authors' contributions

ZQ, SX, and ZM conceived of the study. JT and SX developed the search strategies. ZQ, JT and SX wrote the first draft. TAF and ZL revised the draft. SX and ZM will independently screen potential studies and extract data from the included studies. JW, JK, JT and YL will assess the risk of bias and summarize the evidence. ZM, SX, JK and ZL will address the missing data, if any. ZQ and JT will perform the statistical analysis. ZL and JT will arbitrate in cases of disagreement and ensure the absence of errors. All authors approve the publication of this protocol.

Ethics and dissemination

This protocol will not disseminate any private patient data. The results of this review will be disseminated through peer-reviewed publication.

Competing interests: None.

Funding: None.

For peer review only - http://bmjopen.bmj.com/site/about/guidelines.xhtml

For peer review only

Reference

1. Headache Classification Committee of the International Headache Society (IHS). The International Classification of Headache Disorders, 3rd edition (beta version). Cephalalgia. 2013;33:629-808.

2. Nurmikko TJ. Trigeminal neuralgia-pathophysiology, diagnosis and current treatment. Bja British Journal of Anaesthesia. 2001;87:117-132.

3. Meller D, Obermann M, Yoon MS, Poitz F, Hansen N, Slomke MA. Prevalence of trigeminal neuralgia and persistent idiopathic facial pain: a population-based study. Cephalalgia. 2011;31:1542-1548.

4. MacDonald BK, Cockerell OC, Sander JW, Shorvon SD. The incidence and lifetime prevalence of neurological disorders in a prospective community-based study in the UK. Brain. 2000;123:665-676.

5. Devor M, Amir R, Rappaport ZH. Pathophysiology of trigeminal neuralgia: the ignition hypothesis. Clin J Pain. 2002;18:4-13.

6. Danielle DD, Hodaie M, Davis KD. Structural magnetic resonance imaging can identify trigeminal system abnormalities in classical trigeminal neuralgia. Front Neuroanat. 2016;10:95.

7. Katusic S, Beard CM, Bergstralh E, Kurland LT. Incidence and clinical features of trigeminal neuralgia, Rochester, Minnesota, 1945-1984. Ann Neurol. 1990;27:89-95.

8. Gronseth G, Cruccu G, Alksne J, et al. Practice parameter: the diagnostic evaluation and treatment of trigeminal neuralgia (an evidence-based review): report of the Quality Standards Subcommittee of the American Academy of Neurology and the European Federation of Neurological Societies. Neurology. 2008;71:1183-1190.

9. Campbell FG, Graham JG, Zilkha KJ. Clinical trial of carbazepine (tegretol) in trigeminal neuralgia. J Neurol Neurosurg Psychiatry. 1966;29:265-267.

10. Fromm GH, Terrence CF, Chattha AS. Baclofen in the treatment of trigeminal neuralgia: double-blind study and long-term follow-up. Ann Neurol. 1984;15:240-244.

11. Zakrzewska JM, Lopez BC, Kim SE, Coakham HB. Patient reports of satisfaction after microvascular depression and partial sensory rhizotomy fo trigeminal neuralgia. Neurosurgery. 2005;56:1304-1311.

12. A Nanda, V Javalkar, S Zhang, O Ahmed. Long term efficacy and patient satisfaction of microvascular decompression and gamma knife radiosurgery for trigeminal neuralgia. Journal of Clinical Neuroscience 2015;22:818-822.

13. Linskey ME, Ratanatharathorn V, Peñagaricano J. A prospective cohort study of microvascular decompression and Gamma Knife surgery in patients with trigeminal. J Neurosurg. 2008;109 Suppl:160-172.

14. Gronseth G, Cruccu G, Alksne J, et al. Practice parameter: the diagnostic evaluation and treatment of trigeminal neuralgia (an evidence-based review): report of the Quality Standards Subcommittee of the American Academy of Neurology and the European Federation of

For peer review only - http://bmjopen.bmj.com/site/about/guidelines.xhtml

Neurological Societies. Neurology. 2008;71:1183-1190.

15. Zakrzewska JM, Thomas DG. Patient's assessment of outcome after three surgical procedures for the management of trigeminal neuralgia. Acta Neurochir (Wien). 1993;122:225-230.

16. Sindrup SH, Jensen TS.. Pharmacotherapy of trigeminal neuralgia. Clin J Pain 2002;18:22-27.

17. Wiffen PJ, McQuay HJ, Moore RA. Carbamazepine for acute and chronic pain. Cochrane Database Syst Rev. 2005;(3):CD005451.

18. Cruccu G, Gronseth G, Alksne J, et al. AAN-EFNS guidelines on trigeminal neuralgia management. Eur J Neurol. 2008;15:1013-1028.

19. Zakrzewska JM, Chaudhry Z, Nurmikko TJ, et al. Lamotrigine (Lamictal) in refractory trigeminal neuralgia: results from a double-blind placebo controlled crossover trial. Pain. 1997;73:223-230.

20. Court JE, Kase CS. Treatment of tic douloureux with a new anticonvulsant (clonazepam). J Neurol Neurosurg Psychiatry. 1976;39:297-299.

21. Yuan M, Zhou HY, Xiao ZL, et al. Efficacy and Safety of Gabapentin vs. Carbamazepine in the Treatment of Trigeminal Neuralgia: A Meta-Analysis. Pain Pract. 2016;16:1083-1091

22. Obermann M, Yoon MS, Sensen K, Maschke M, Diener HC, Katsarava Z. Efficacy of pregabalin in the treatment of trigeminal neuralgia. Cephalalgia. 2008;28:174-181.

23. Peiris JB, Perera GL, Devendra SV, Lionel ND. Sodium valproate in trigeminal neuralgia. Med J Aust. 1980;2:278.

24. McQuay H, Carroll D, Jadad AR, et al. Anticonvulsant drugs for management of pain: a systematic review. BMJ. 1995;311:1047-1052.

25. Wiffen PJ, Rees J. Lamotrigine for acute and chronic pain. Cochrane Database Syst Rev. 2007;(2):CD006044.

26. Wiffen PJ, McQuay HJ, Rees J, Moore RA. Gabapentin for acute and chronic pain. Cochrane Database Syst Rev. 2005;(3):CD005452.

27. Wiffen PJ, Derry S, Moore RA, McQuay HJ. Carbamazepine for acute and chronic pain in adults. Cochrane Database Syst Rev. 2011;(1):CD005451.

28. Moore RA, Straube S, Wiffen PJ, Derry S, McQuay HJ. Pregabalin for acute and chronic pain in adults. Cochrane Database Syst Rev. 2009;(3):CD007076.

29. Caldwell DM, Ades AE, Higgins JP. Simultaneous comparison of multiple treatments: combining direct and indirect evidence. BMJ. 2005;331:897-900.

30. Cipriani A, Higgins JP, Geddes JR, Salanti G. Conceptual and technical challenges in network meta-analysis. Ann Intern Med. 2013;159:130-137.

31. Cipriani A, Furukawa TA, Salanti G, et al. Comparative efficacy and acceptability of 12 new-generation antidepressants: a multiple-treatments meta-analysis. Lancet. 2009;373:746-758.

32. Shamseer L, Moher D, Clarke M, et al. Preferred reporting items for systematic review and meta-analysis protocols (PRISMA-P) 2015: elaboration and explanation. BMJ. 2015;349:g7647.

33. Hutton B, Salanti G, Caldwell DM, et al. The PRISMA Extension Statement for Reporting of

For peer review only - http://bmjopen.bmj.com/site/about/guidelines.xhtml

Systematic Reviews Incorporating Network Meta-analyses of Health Care Interventions: Checklist and Explanations. Ann Intern Med. 2015;162:777-784.

34. Merskey H, Bogduk N. Classification of chronic pain. Descriptions of chronic pain syndromes and definitions of pain terms, IASP Press, Seattle 1994, pp. 59-71.

35. Zakrzewska JM. Diagnosis and differential diagnosis of trigeminal neuralgia. Clin J Pain. 2002;18:14-21.

36. Dworkin RH, Turk DC, Farrar JT, et al. Core outcome measures for chronic pain clinical trials: IMMPACT recommendations. Pain. 2005;113:9-19.

37. Ware JE Jr, Sherbourne CD. The MOS 36-item short-form health survey (SF-36). I. Conceptual framework and item selection. Med Care. 1992;30:473-483.

38. Qin Z, Li B, Wu J, et al. Acupuncture for chronic diarrhea in adults: protocol for a systematic review. Medicine (Baltimore). 2017;94:e5952.

39. Higgins JPT, Altman DG, Sterne JAC (editors). Chapter 8: Assessing risk of bias in included studies. In: Higgins JPT, Green S(editors). Cochrane Handbook for Systematic Reviews of Intervention Version 5.1.0 [updated March 2011]. The Cochrane Collaboration, 2011. Available from www.cochrane-handbook.org.

40. Salanti G, Del Giovane C, Chaimani A, et al. Evaluating the quality of evidence from a network meta-analysis. PLoS ONE. 2014;9:e99682.

41. Furukawa TA, Cipriani A, Barbui C, Brambilla P, Watanabe N. Imputing response rates from means and standard deviations in meta-analyses. Int Clin Psychopharmacol. 2005;20:49-52.

42. Chaimani A, Higgins JPT, Mavridis D, Spyridonos P, Salanti G. Graphical tools for network meta-analysis in STATA. PLoS One. 2013;8:e76654.

43. Higgins JP, Thompson SG, Deeks JJ, Altman DG. Measuring inconsistency in meta-analyses. BMJ. 2003;327:557-560.

44. Baker SG, Kramer BS. The transitive fallacy for randomized trials: if A bests B and B bests C in separate trials, is A better than C? BMC Med Res Methodol. 2002;2:13.

45. White IR, Barrett JK, Jackson D, et al. Consistency and inconsistency in network meta-analysis: model estimation using multivariate meta-regression. Res Synth Methods. 2012;3:111-125

46. Higgins JPT, Jackson D, Barrett JL, et al. Consistency and inconsistency in network meta-analysis: concepts and models for multi-arm studies. Res Synth Methods. 2012;3:98-110

47. Dias S, Welton NJ, Caldwell DM, et al. Checking consistency in mixed treatment comparison meta-analysis. Stat Med. 2010;29:932-944

48. Lu G, Ades AE. Assessing evidence inconsistency in mixed treatment comparisions. J Am Stat Assoc. 2006;101:447-459.

49. Veroniki AA, Vasiliadis HS, Higgins JP, Salanti G. Evaluation of inconsistency in networks of interventions. Int J Epidemiol. 2013;42:332-345.

For peer review only - http://bmjopen.bmj.com/site/about/guidelines.xhtml

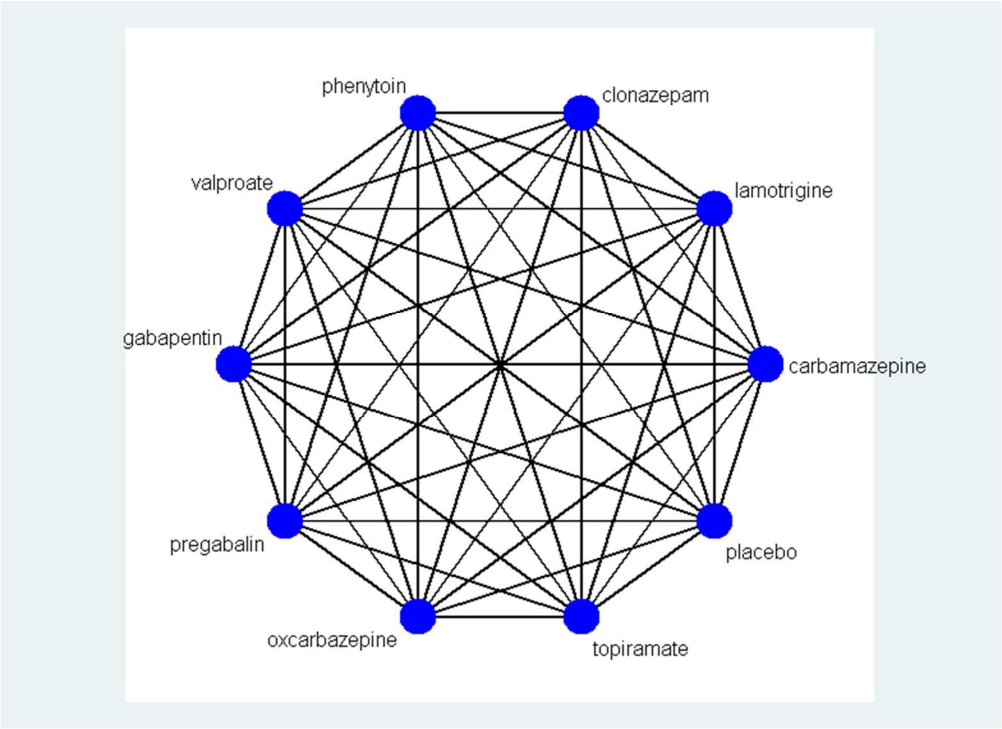

Network plot of all possible direct comparisons between the eligible interventions.

276x201mm (72 x 72 DPI)

For peer review only

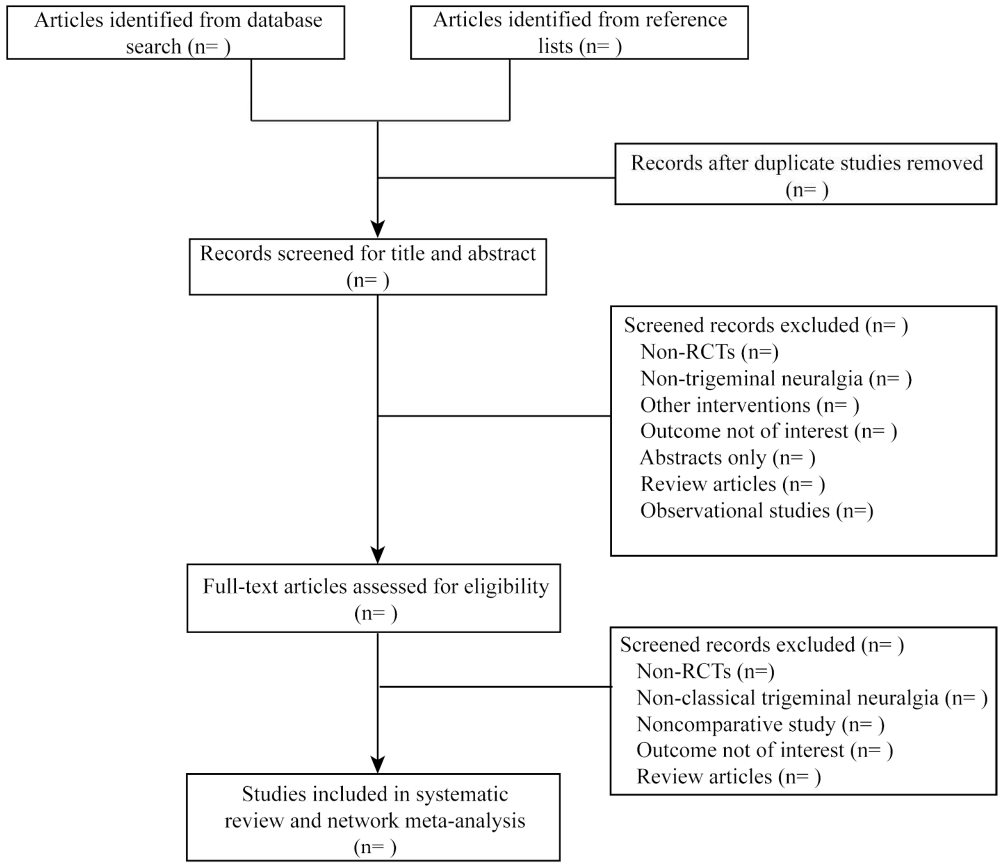

FIGURE 1. PRISMA flow chart.

PRISMA flow chart.

192x225mm (300 x 300 DPI)

PRISMA-P (Preferred Reporting Items for Systematic review and Meta-Analysis Protocols) 2015 checklist: recommended items to address in a systematic review protocol*

Section and topic	Item No	Checklist item
**ADMINISTRATIVE INFORMATION**		
Title:		
Identification	1a	Identify the report as a protocol of a systematic review Page 1
Update	1b	If the protocol is for an update of a previous systematic review, identify as such NA
Registration	2	If registered, provide the name of the registry (such as PROSPERO) and registration number Page 2
Authors:		
Contact	3a	Provide name, institutional affiliation, e-mail address of all protocol authors; provide physical mailing address of corresponding author Page 1
Contributions	3b	Describe contributions of protocol authors and identify the guarantor of the review Page 11
Amendments	4	If the protocol represents an amendment of a previously completed or published protocol, identify as such and list changes; otherwise, state plan for documenting important protocol amendments Page 11
Support:		
Sources	5a	Indicate sources of financial or other support for the review Page 11
Sponsor	5b	Provide name for the review funder and/or sponsor Page 11
Role of sponsor or funder	5c	Describe roles of funder(s), sponsor(s), and/or institution(s), if any, in developing the protocol Page 11
**INTRODUCTION**		
Rationale	6	Describe the rationale for the review in the context of what is already known Page 3
Objectives	7	Provide an explicit statement of the question(s) the review will address with reference to participants, interventions, comparators, and outcomes (PICO) Page 4
**METHODS**		
Eligibility criteria	8	Specify the study characteristics (such as PICO, study design, setting, time frame) and report characteristics (such as years considered, language, publication status) to be used as criteria for eligibility for the review Page 4
Information sources	9	Describe all intended information sources (such as electronic databases, contact with study authors, trial registers or other grey literature sources) with planned dates of coverage Page 5
Search strategy	10	Present draft of search strategy to be used for at least one electronic database, including planned limits, such that it could be repeated Page 6
Study records:		
Data management	11a	Describe the mechanism(s) that will be used to manage records and data throughout the review Page 6-7

For peer review only

Selection process	11b	State the process that will be used for selecting studies (such as two independent reviewers) through each phase of the review (that is, screening, eligibility and inclusion in meta-analysis) Page 6-7
Data collection process	11c	Describe planned method of extracting data from reports (such as piloting forms, done independently, in duplicate), any processes for obtaining and confirming data from investigators Page 6-7
Data items	12	List and define all variables for which data will be sought (such as PICO items, funding sources), any pre-planned data assumptions and simplifications Page 6-7
Outcomes and prioritization	13	List and define all outcomes for which data will be sought, including prioritization of main and additional outcomes, with rationale Page 5-6
Risk of bias in individual studies	14	Describe anticipated methods for assessing risk of bias of individual studies, including whether this will be done at the outcome or study level, or both; state how this information will be used in data synthesis Page 7
Data synthesis	15a	Describe criteria under which study data will be quantitatively synthesised Page 7-8
	15b	If data are appropriate for quantitative synthesis, describe planned summary measures, methods of handling data and methods of combining data from studies, including any planned exploration of consistency (such as I^2, Kendall's τ) Page 8
	15c	Describe any proposed additional analyses (such as sensitivity or subgroup analyses, meta-regression) Page 9-10
	15d	If quantitative synthesis is not appropriate, describe the type of summary planned Page 9-10
Meta-bias(es)	16	Specify any planned assessment of meta-bias(es) (such as publication bias across studies, selective reporting within studies) Page 7
Confidence in cumulative evidence	17	Describe how the strength of the body of evidence will be assessed (such as GRADE) Page 7

*** It is strongly recommended that this checklist be read in conjunction with the PRISMA-P Explanation and Elaboration (cite when available) for important clarification on the items. Amendments to a review protocol should be tracked and dated.** **The copyright for PRISMA-P (including checklist) is held by the PRISMA-P Group and is distributed under a Creative Commons Attribution Licence 4.0.**

From: Shamseer L, Moher D, Clarke M, Ghersi D, Liberati A, Petticrew M, Shekelle P, Stewart L, PRISMA-P Group. Preferred reporting items for systematic review and meta-analysis protocols (PRISMA-P) 2015: elaboration and explanation. BMJ. 2015 Jan 2;349(jan02 1):g7647.

For peer review only

PubMed

Patient

#1 "Trigeminal Neuralgia"[Mesh]

#2 Trigeminal Neuralgia*[Title/Abstract] OR Trifacial Neuralgia*[Title/Abstract] OR Fothergill Disease[Title/Abstract] OR Tic Douloureux[Title/Abstract] OR Epileptiform Neuralgia*[Title/Abstract] OR trigeminus neuralgia[Title/Abstract] OR prosopalgia[Title/Abstract] OR prosoponeuralgia[Title/Abstract] OR trigeminal nerve neuralgia[Title/Abstract] OR trigeminal nerve neuropathy[Title/Abstract] OR trigeminal neuropathy[Title/Abstract] OR trigeminus nerve neuralgia[Title/Abstract] OR trigeminus nerve neuropathy[Title/Abstract]

#3 #1 OR #2

RCT

#4 "Clinical Trials, Phase II as Topic"[Mesh] OR "Clinical Trials, Phase III as Topic"[Mesh] OR "Clinical Trials, Phase IV as Topic"[Mesh] OR "Controlled Clinical Trials as Topic"[Mesh] OR "Randomized Controlled Trials as Topic"[Mesh] OR "Intention to Treat Analysis"[Mesh] OR "Pragmatic Clinical Trials as Topic"[Mesh] OR "Clinical Trials, Phase II"[Publication Type] OR "Clinical Trials, Phase III"[Publication Type] OR "Clinical Trials, Phase IV"[Publication Type] OR "Controlled Clinical Trials"[Publication Type] OR "Randomized Controlled Trials"[Publication Type] OR "Pragmatic Clinical Trials as Topic"[Publication Type] OR "Single-Blind Method"[Mesh] OR "Double-Blind Method"[Mesh]

#5 random*[Title/Abstract] OR blind*[Title/Abstract] OR singleblind*[Title/Abstract] OR doubleblind*[Title/Abstract] OR trebleblind*[Title/Abstract] OR tripleblind*[Title/Abstract]

#6 #4 OR #5

#7 #3 AND #6

EMBASE.com

Patient

#1 'trigeminus neuralgia'/exp

#2 'trigeminal neuralgias':ab,ti OR 'trifacial neuralgias':ab,ti OR 'trigeminal neuralgia':ab,ti OR 'trifacial neuralgia':ab,ti OR 'fothergill disease':ab,ti OR 'tic douloureux':ab,ti OR 'epileptiform neuralgia':ab,ti OR 'epileptiform neuralgias':ab,ti OR 'trigeminus neuralgia':ab,ti OR prosopalgia:ab,ti OR prosoponeuralgia:ab,ti OR 'trigeminal nerve neuralgia':ab,ti OR 'trigeminal nerve neuropathy':ab,ti OR 'rigeminal neuropathy':ab,ti OR 'trigeminus nerve neuralgia':ab,ti OR 'trigeminus nerve neuropathy':ab,ti

#3 #1 OR #2

RCT

#4 'multicenter study (topic)'/exp OR 'phase 2 clinical trial (topic)'/exp OR 'phase 3 clinical trial

For peer review only

(topic)'/exp OR 'phase 4 clinical trial (topic)'/exp OR 'controlled clinical trial (topic)'/exp OR 'randomized controlled trial (topic)'/exp OR 'single blind procedure'/exp OR 'double blind procedure'/exp

#5 random*:ab,ti OR blind*:ab,ti OR singleblind*:ab,ti OR doubleblind*:ab,ti OR trebleblind*:ab,ti OR tripleblind*:ab,ti

#6 #4 OR #5

#7 #3 AND #6

Cochrane Library

#1 MeSH descriptor: [Trigeminal Neuralgia] explode all trees

#2 Trigeminal Neuralgia*:ti,ab,kw or Trifacial Neuralgia*:ti,ab,kw or Fothergill Disease:ti,ab,kw or Tic Douloureux:ti,ab,kw or Epileptiform Neuralgia*:ti,ab,kw or trigeminus neuralgia:ti,ab,kw or prosopalgia:ti,ab,kw or prosoponeuralgia:ti,ab,kw or trigeminal nerve neuralgia:ti,ab,kw or trigeminal nerve neuropathy:ti,ab,kw or trigeminal neuropathy:ti,ab,kw or trigeminus nerve neuralgia:ti,ab,kw or trigeminus nerve neuropathy:ti,ab,kw

#3 #1 or #2

For peer review only

BMJ Open

Comparative Efficacy and Acceptability of Antiepileptic Drugs for Classical Trigeminal Neuralgia: A Bayesian Network Meta-Analysis Protocol

Journal:	*BMJ Open*
Manuscript ID	bmjopen-2017-017392.R1
Article Type:	Protocol
Date Submitted by the Author:	21-Jul-2017

Complete List of Authors:	Qin, Zongshi; Guang'anmen Hospital, China Academy of Chinese Medical Sciences, Department of Acupuncture and Neurology<br>Xie, Shang; Peking University School and Hospital of Stomatology, Department of Oral and Maxillofacial Surgery<br>Mao, Zhi; Chinese People's Liberation Army General Hospital, Department of Critical Care Medicine<br>Liu, Yan; China Academy of Chinese Medical Sciences, Data Centre of Traditional Chinese Medicine<br>WU, Jiani; Guang'anmen Hospital, China Academy of Chinese Medical Sciences, Department of Acupuncture and Neurology<br>Furukawa, Toshi; Kyoto University, Graduate School of Medicine and School of Public Health<br>Kwong, Joey; Taipei Medical University, Cochrane Taiwan; National Center for Child Health and Development, Department of Health Policy & Department of Clinical Epidemiology<br>Tian, Jinhui; Evidence based medicine center,<br>Liu, Zhishun; Guang'anmen Hospital, China Academy of Chinese Medical Sciences, Department of Acupuncture and Neurology

**Primary Subject Heading**:	Evidence based practice
Secondary Subject Heading:	Neurology
Keywords:	antiepileptic drugs, trigeminal neuralgia, network meta-analysis, systematic review, protocol

SCHOLARONE™ Manuscripts

Comparative Efficacy and Acceptability of Antiepileptic Drugs for Classical Trigeminal Neuralgia: A Bayesian Network Meta-Analysis Protocol

Zongshi Qin,[1] Shang Xie[2], Zhi Mao[3], Yan Liu[4], Jiani Wu[1], Toshi A Furukawa[5], Joey S.W. Kwong[6,7], Jinhui Tian[8], Zhishun Liu[1]

[1] Department of Acupuncture and Neurology, Guang'anmen Hospital, China Academy of Chinese Medical Sciences, Beijing, China

[2] Department of Oral and Maxillofacial Surgery, Peking University School and Hospital of Stomatology, Beijing, China

[3] Department of Critical Care Medicine, Chinese People's Liberation Army General Hospital, Beijing, China

[4] Data Centre of Traditional Chinese Medicine, China Academy of Chinese Medical Sciences, Beijing, China

[5] Department of Health Promotion and Human Behavior, Kyoto University Graduate School of Medicine/School of Public Health, Kyoto, Japan

[6] Cochrane Taiwan, Taipei Medical University, Taipei, Taiwan

[7] Department of Health Policy & Department of Clinical Epidemiology, National Center for Child Health and Development, Tokyo, Japan

[8] Evidence-based Medicine Center, Lanzhou University, Lanzhou, China

ZQ, SX and ZM contributed equally to this work.

Correspondence to: Jinhui Tian, email: tianjh@lzu.edu.cn; Zhishun Liu, email: liuzhishun@aliyun.com

Keywords: antiepileptic drugs; trigeminal neuralgia; network meta-analysis; systematic review; protocol

For peer review only - http://bmjopen.bmj.com/site/about/guidelines.xhtml

ABSTRACT

Introduction Trigeminal neuralgia (TN) affects 4 to 28.9/100,000 people worldwide, and antiepileptic drugs such as carbamazepine and oxcarbazepine are the first-line treatment options. However, the efficacy and safety of other antiepileptic drugs remain unclear due to insufficient direct comparisons.

Objective To compare the efficacy and acceptability of all currently available antiepileptic agents for the treatment of patients with classical TN.

Methods We will search the PubMed, EMBASE, Cochrane Library, and Web of Science databases for unpublished or undergoing research listed in registry platforms. We will include all randomized controlled trials comparing two different antiepileptic drugs or one antiepileptic drug with placebo in patients with classic TN. The primary outcomes will be the proportion of responders and the number of subjects who drop out during the treatment. The secondary outcomes include the two primary outcomes but set in the follow-up period, changes in the self-reporting assessment scale for neuralgia, and quality of life assessment. In terms of network meta-analysis, we will fit our model in a Bayesian framework using WinBUGS (Medical Research Council's Biostatistics Unit, Cambridge, UK). To confirm the results, we will also conduct analyses using Stata (Version 13.0; Stata Corporation, College Station, Texas, USA) and compare the differences between the two platforms.

Ethics and dissemination This protocol will not disseminate any private patient data. The results of this review will be disseminated through peer-reviewed publication.

Protocol registration for this systematic review (registration number): PROSPERO (CRD: 42016048640).

Strengths and limitations of this study

- To the best of the authors' knowledge, this study will be the first network meta-analysis that assess the comparative efficacy and acceptability of all the available antiepileptic drugs for the classical trigeminal neuralgia.
- This study will be performed by Bayesian framework, which enables us to estimate the probability for each intervention to be the best for each outcome.
- Owing to the language barrier, the amount of included trials might be potentially limited.

For peer review only

For peer review only - http://bmjopen.bmj.com/site/about/guidelines.xhtml

Introduction

Classical trigeminal neuralgia (TN), a chronic pain disorder described as one of the most severe pains one can suffer, is characterized by paroxysms of unilateral, electric shock-like, and severe pain along the trigeminal nerve divisions.[1,2] It affects lifestyle because it can be triggered by common activities such as eating, talking, shaving and brushing teeth. The wind, chewing and talking also aggravate the condition in many patients.[2] It is estimated that approximately 4 to 28.9 per 100,000 people worldwide suffer from TN, and the number affected tends to be higher among women at all ages and even increases with age.[3,4]

At present, the cause of TN remains unclear.[5,6] One hypotheses is that the trigeminal nerve becomes compressed at the root entry zone by cerebral vessels.[7] Owing to the contradictory etiology and poorly understood pathophysiological mechanisms underlying TN, a variety of therapeutic and surgical approaches have been developed to alleviate the associated pain and improve the quality of life in patients with classical TN.[8-10] Although many patients have obtained excellent outcomes from surgery, many others do not experience any pain relief.[11,12] Furthermore, the currently available surgical procedures are associated with various complications, particularly sensory loss in the trigeminal nerve territory, anesthesia dolorosa and, rarely, ipsilateral hearing loss, depending on the technique.[13,14]

As such, pharmacological measures to improve clinical outcomes are needed. The most commonly used option is antiepileptic drugs, with phenytoin being the first drug used for classical TN with positive effect.[15] Carbamazepine can reduce both the frequency and intensity of painful paroxysms and was first introduced by the US Food and Drug Administration (FDA); however, its efficacy is compromised by poor tolerability.[16] Oxcarbazepine, a derivative of carbamazepine, is often used as an initial treatment for classical TN and has more favorable properties than carbamazepine related to its increased efficacy in epilepsy, greater tolerability and decreased potential for drug interactions.[17] Lamotrigine has also been reported as an effective add-on therapy,[18] whereas little evidence supports that other antiepileptic drugs such as clonazepam, gabapentin, pregabalin and valproate have a beneficial effect.[19-22] However, many of the studies are old with limited methodology, and were assessed as low GRADE scores.[23]

To date, several systematic reviews have investigated the comparative efficacy and safety of antiepileptic drugs.[20,24-28] However, previous systematic reviews have only considered pair-wise

For peer review only - http://bmjopen.bmj.com/site/about/guidelines.xhtml

evidence from head-to-head comparisons and have thus failed to assess the comparative efficacy and acceptability of all available antiepileptic drugs. Thus, it is difficult to determine the best treatments for relieving pain with minimal adverse effects. In the present study, we will apply network meta-analysis to integrate direct and indirect comparisons,[29,30] which could be used not only to strengthen inferences concerning the efficacy and acceptability of treatments but also to rank the efficacy and acceptability of antiepileptic drugs accordingly.[31]

The objectives of this systematic review and network meta-analysis are 1) to compare all currently available antiepileptic drugs in terms of efficacy and acceptability in classical TN treatment and 2) to determine which drug achieves the best balance between efficacy and adverse effects. The results of this study will augment findings based on current pair-wise meta-analyses and are expected to provide important information to support clinical practice and health policy decisions.

METHODS

This protocol will be conducted in accordance with the reporting guidance provided in the Preferred Reporting Items for Systematic Reviews and Meta-Analysis Protocols (PRISMA-P) statement and Checklist of Items to Include When Reporting a Systematic Review Involving a Network Meta-analysis.[32,33] The protocol is registered in PROSPERO (CRD: 42016048640). This study will not involve any private patient data, ethical approval was waived. (supplemental file 1 represents the PRISMA-P checklist)

Eligibility criteria

Study types

We will include randomized controlled trials (RCTs) comparing one antiepileptic drug with another antiepileptic drug as monotherapy or placebo for the treatment of TN. Quasi-randomized controlled trails allocating participants according to birth date or the consequences of enrollment will be excluded. The minimum duration for RCT inclusion was set at 4 weeks. Trials with more than a two-arm design will be considered only if the available data meet the criteria for an intervention. For trials with a crossover design, data will only be extracted from the first randomization period.

For peer review only - http://bmjopen.bmj.com/site/about/guidelines.xhtml

Participant characteristics

Only trials that enrolled participants with a diagnosis of classical TN according to standardized criteria such as the classification of the International Headache Society (IHS) of International Classification of Headache Disorders will be sought.[1,34] For studies using other extensive criteria for the diagnosis of classical TN, detailed diagnostic criteria must be reported (such as history or characteristics that have been confirmed by CT or MRI).[35] Studies examining symptomatic TN patients will not be included. Participants with comorbid conditions such as anxiety, depression, epilepsy or other medical conditions will also not be eligible for inclusion. No limitations will be imposed for age, sex, or nationality.

Intervention types

We plan to include the following antiepileptic drugs: carbamazepine, lamotrigine, clonazepam, phenytoin, valproate, gabapentin, pregabalin, oxcarbazepine and topiramate. In addition to these antiepileptic drugs, we will also obtain information about interventions of interest from either pair-wise RCTs or placebo-controlled trails, as some RCTs design a placebo-controlled arm as the comparator. Figure 1 illustrates the network plot of all possible direct comparisons between the eligible interventions.

Outcome measures

Studies reporting one of the following will be included.

Primary outcome

The primary objective of this review is to assess the efficacy and acceptability of antiepileptic drugs for classical TN; therefore, the following two outcomes will be used as the primary outcome.

1. The proportion of responders to a self-reporting assessment scale for neuralgia. A responder was defined as a subject who obtained a ≥ 50% pain reduction score from baseline to endpoint (4–12 weeks) or a subject who obtained a pain reducing score of no less than the minimal clinically important difference (MCID). Pain scores will be extracted based on the visual analogue score (VAS), numerical rating score (NRS), or any other validated scale for the assessment of overall TN symptoms when available.[36]

For peer review only - http://bmjopen.bmj.com/site/about/guidelines.xhtml

2. Treatment acceptability is defined as the proportion of patients who have intervention related adverse events during the 4 to 12 weeks.

Secondary outcomes

1. The proportion of responders with ≥ 50% pain reduction on a self-reporting assessment scale for neuralgia from baseline to endpoint after follow-up.

2. The change in pain symptoms of TN from baseline to endpoint (4–12 weeks) measured based on the VAS, NRS, or any other validated scale for the assessment of overall TN symptoms when available.

3. The change in pain symptoms of TN from baseline to endpoint after follow-up.

4. The quality of life based on measurement with a validated scale, such as the Short Form 36 Health Survey questionnaire (SF-36).[37]

Search strategy

We will identify RCTs through a comprehensive, systematic literature search primarily utilizing the PubMed, EMBASE, Cochrane Library, and Web of Science databases. As publication bias caused by insufficient unpublished data can significantly bias the comparative efficacy results of network meta-analysis and modify rankings, we will also perform searches for unpublished or ongoing trials using System for information on Grey Literature in Europe (SIGLE) as well as other registry platforms, such as Clinicaltrials.gov and the International Clinical Trials Registry Platform. Prior to completing this review, we will perform an additional search of each database and registration platform to guarantee that the most recent studies are included. We will use medical subject headings and text words related to ‘trigeminal neuralgia’ and ‘randomized controlled trial’ for the literature search. In addition, the reference lists of previous systematic reviews will be examined to ensure the quantity and accuracy of the included studies. The search strategy will be developed by JT and ZL, we anticipate that the aforementioned databases will be searched at 30th Sept., 2017. (supplemental file 2 represents the search strategies for PubMed, EMBASE and Cochrane Library)

Data collection process

Two authors (SX and ZM) will scan the titles and abstracts of the trials after duplicated records

For peer review only - http://bmjopen.bmj.com/site/about/guidelines.xhtml

For peer review only

have been excluded using EndNote X7 (Thomson Reuters, New York, NY). The scanning will be performed using EndNote, and all trials will be allocated to the following five groups: inclusion group, non-patient group, intervention group, outcome group, and awaiting group. A prior data collection process will be conducted using an electro-table created with Excel software, which has been used in our previous study.[38] The table will consist of four sheets, including general information (author list, publication year, and journal), characteristics of included trials (diagnostic criteria, age range, study drugs, and dose range), the risk of bias assessed using the Cochrane risk of bias tool, and outcome data extraction (number of participants who responded to treatment and the number who dropped out during the treatment). All original data will be submitted as an attachment. A flow chart illustrating this design is presented in Figure 2.

Quality assessment

Two authors (JW and YL) will use the Cochrane risk of bias tool to assess the risk of bias of eligible studies, covering randomization, concealment allocation, blinding and other biases.[39] As inadequate concealment could potentially fail the randomization test, two independent review authors will pay particular attention to the adequacy of random allocation concealment and blinding. The other sources of bias will be assessed while considering sample size calculation method, diagnostic criteria, reporting of withdrawals and follow-up. Two authors (JK and JT) will assess the quality of evidence using Grading of Recommendations Assessment, Development and Evaluation (GRADE) framework, covering study limitations, inconsistency, indirectness, imprecision and publication bias.[40] The methods for rating the quality of direct comparisons are the same to the methods used in traditional meta-analysis, and following steps will be used in the whole assessment procedure: 1) presenting direct and indirect effect estimates; 2) rating the quality of direct and indirect estimates; 3) presenting the results of network meta-analysis; 4) rating the quality of network meta-analysis effect estimates.

Dealing with missing data

To obtain missing data, we will initially contact the senior or corresponding author. If no one responds, we will estimate the missing data as follows. For studies failing to report the number of responding patients after treatment, instead of providing the mean and standard deviation, we will

For peer review only - http://bmjopen.bmj.com/site/about/guidelines.xhtml

calculate the number of responding patients employing a validated imputation method.[41] In addition, we will also estimate missing data from graphs when possible. For trials that cannot be extracted or estimated, the available data will be excluded, and the reason for exclusion will be reported.

Statistical analysis

The method used for data synthesis will be based on mixed treatment meta-analysis. To examine comparisons, we will use Stata (Version 13.0; Stata Corporation, College Station, Texas, USA) to synthesize data and will present the comparison results if the included studies are sufficient for each pair-wise comparison. We will use a random effects model to combine the data, and the outcomes of continuous and binary variables will be presented as standardized mean differences (SMDs) and odds ratios (ORs) with 95% confidential intervals (CIs). For indirect comparisons, a random effects model network meta-analysis will be developed in a Bayesian framework using Markov chain Monte Carlo simulation methods in WinBUGS (Medical Research Council's Biostatistics Unit, Cambridge, UK). The results of network meta-analysis will use the arm-based parameterization for random effects model.[42] This will enable us to estimate the best probability for each intervention for each positive outcome, given the results of the multiple-treatment meta-analysis. At least one network focusing on the response rate for pain relief will be constructed, in which a statistically significant difference defined as the null value will not be included in the 95% CI. The Markov chains will be utilized for 50,000 simultaneous iterations based on the data and the description of the proposed distributions for relevant parameters, and the first 10,000 iterations will be discarded to avoid potential impact on the arbitrary value. For continuous outcomes and binary outcomes, the OR and SMD will be presented with the 95% credible interval (CrI). In this process, the Brooks-Gelman-Rubin method will be used to assess the convergence between direct and indirect variances. According to the theory of Brooks and Gelman, if a potential scale reduction factor (PSRF) is less than 1.2, then an approximate convergence has occurred. The PSRF results will be presented graphically using a Brooks-Gelman-Rubin diagnosis plot, if needed. To describe relationships among different treatments, a network plot will be created to show direct comparisons between arms based on different outcomes. To confirm the results, we will also conduct the same network meta-analysis

For peer review only - http://bmjopen.bmj.com/site/about/guidelines.xhtml

using the network package of Stata (http://www.mrc-bsu.cam.ac.uk/IW_Stata/), and the outcome will be compared to that produced using WinBUGS. In addition, the effectiveness of each treatment among all available treatments will be ranked by calculating the OR in order, and plots of the surfaces under the cumulative ranking curves (SUCRAs) will be generated to rank the various treatments for each outcome.[43] We will also present a cluster rank table to synthesize the efficacy and acceptability of each drug (using two primary outcomes). The table will consist of two triangles: the upper right triangle will illustrate the acceptability, and the lower left triangle will illustrate the efficacy.[31]

Assessment of heterogeneity

Heterogeneity, which plays a pivotal role in both standard meta-analysis and network meta-analysis, refers to the degree of disagreement between study-specific treatment effects and constitutes the basis of inconsistency. To test the heterogeneity of each pair-wise comparison, we will use the I^2 statistic.[44]

Assessment of transitivity and similarity

In addition to the heterogeneity assessment using the I^2 statistic, the assumption of transitivity and similarity based on clinical and methodological characteristics will be assessed. It should be noted that it is difficult to identify these effect modifiers using statistical analysis. We will assume that intervention effects are transitive in this network meta-analysis because we will only focus on antiepileptic drugs, and we will investigate similarity based on clinical characteristics, such as antiepileptic drug dose, period of treatment, and severity of pain symptoms at baseline, as well as according to methodological characteristics such as study quality.[45] All these effect modifiers will be judged and reported before the network meta-analysis is conducted.

Assessment of inconsistency

The evaluation and explanation of inconsistency is another basic objective of network meta-analysis. In this context, inconsistency refers to the degree of difference between direct and indirect comparisons and can be evaluated only when a loop exists in the evidence network. This means that inconsistency assessment using a design-by-treatment interaction model cannot be conducted if the structure of this network is a “star network” (i.e., all interventions have a single

For peer review only - http://bmjopen.bmj.com/site/about/guidelines.xhtml

mutual comparator, such as a placebo).[46,47] For such cases, we will test inconsistency using a node-splitting model.[48]

To identify inconsistency among the included trials of the network, we will use Stata, performing the Z test to compare direct and indirect summary effects in specific loops.[49] If there is no inconsistency between loops or designs, we will use a consistency model to calculate the data. For cases of significant incoherence, we will initially look for data extraction errors in loops that present inconsistency and in comparisons with large heterogeneity.[50] After the data have been scrutinized, we will investigate possible sources of inconsistency within clinical and methodological variables suspected of being potential sources of either heterogeneity or incoherence in each comparison-specific group of trials. If an important inconsistency cannot be explained, we will consider avoiding synthesis of the related network.

Additional analyses

To ensure the quality of this review, studies not reporting blinding will be excluded prior to data synthesis because blinding plays a vital important role in the randomized controlled trial. We will assess heterogeneity quantitatively using the I^2 statistic, and if an I^2 value is greater than 50%, then we will explore the source of heterogeneity. We will initially perform sensitivity analysis by excluding trials rated as having a high risk of bias. Additionally, meta-regression or subgroup analysis will be used to explore possible sources of heterogeneity if the number of included trials is sufficient. For network meta-regression, we will use a random effects network meta-regression model to examine potential factors.

Discussion

To the best of our knowledge, no network meta-analyses comparing the use of antiepileptic drugs for treatment of classical TN have been conducted to date. Previous systematic reviews have compared only a single drug to other types of drug or therapy.[20,24-28] This makes it difficult to obtain a clear understanding of the effectiveness of the various different conservative treatments for this disorder. Network meta-analysis can be used to perform indirect comparisons and allows parameters for direct and indirect comparisons to be synthesized. To ensure the quantity and quality of the potentially included RCTs, we will perform an extensive literature search and

For peer review only - http://bmjopen.bmj.com/site/about/guidelines.xhtml

predefine rigorous inclusion criteria. Besides, we will assess the quality of evidence with the GRADE framework. Although a ranking of the included interventions will be generated, with the exception of findings, the quality of evidence should also be considered. We hope that the results of this review help clinicians make more accurate treatment decisions and promote additional research into conservative treatments for classical TN.

For peer review only

For peer review only - http://bmjopen.bmj.com/site/about/guidelines.xhtml

Amendments

If it is necessary to update this protocol, we will update this protocol in the future. We will submit the original protocol, final protocol and summary of changes as a supplement.

Authors' contributions

ZQ, SX, and ZM conceived of the study. JT and SX developed the search strategies. ZQ, JT and SX wrote the first draft. TAF and ZL revised the draft. SX and ZM will independently screen potential studies and extract data from the included studies. JW, JK, JT and YL will assess the risk of bias and summarize the evidence. ZM, SX, JK and ZL will address the missing data, if any. ZQ and JT will perform the statistical analysis. ZL and JT will arbitrate in cases of disagreement and ensure the absence of errors. All authors approve the publication of this protocol.

Ethics and dissemination

This protocol will not disseminate any private patient data. The results of this review will be disseminated through peer-reviewed publication.

Competing interests: None.

Funding: None.

For peer review only

Reference

1. Headache Classification Committee of the International Headache Society (IHS). The International Classification of Headache Disorders, 3rd edition (beta version). Cephalalgia. 2013;33:629-808.

2. Zakrzewska JM, Linskey ME. Trigeminal neuralgia. BMJ. 2015;350:h1238

3. Meller D, Obermann M, Yoon MS, Poitz F, Hansen N, Slomke MA. Prevalence of trigeminal neuralgia and persistent idiopathic facial pain: a population-based study. Cephalalgia. 2011;31:1542-1548.

4. MacDonald BK, Cockerell OC, Sander JW, Shorvon SD. The incidence and lifetime prevalence of neurological disorders in a prospective community-based study in the UK. Brain. 2000;123:665-676.

5. Devor M, Amir R, Rappaport ZH. Pathophysiology of trigeminal neuralgia: the ignition hypothesis. Clin J Pain. 2002;18:4-13.

6. Danielle DD, Hodaie M, Davis KD. Structural magnetic resonance imaging can identify trigeminal system abnormalities in classical trigeminal neuralgia. Front Neuroanat. 2016;10:95.

7. Katusic S, Beard CM, Bergstralh E, Kurland LT. Incidence and clinical features of trigeminal neuralgia, Rochester, Minnesota, 1945-1984. Ann Neurol. 1990;27:89-95.

8. Gronseth G, Cruccu G, Alksne J, et al. Practice parameter: the diagnostic evaluation and treatment of trigeminal neuralgia (an evidence-based review): report of the Quality Standards Subcommittee of the American Academy of Neurology and the European Federation of Neurological Societies. Neurology. 2008;71:1183-1190.

9. Campbell FG, Graham JG, Zilkha KJ. Clinical trial of carbazepine (tegretol) in trigeminal neuralgia. J Neurol Neurosurg Psychiatry. 1966;29:265-267.

10. Fromm GH, Terrence CF, Chattha AS. Baclofen in the treatment of trigeminal neuralgia: double-blind study and long-term follow-up. Ann Neurol. 1984;15:240-244.

11. Zakrzewska JM, Lopez BC, Kim SE, Coakham HB. Patient reports of satisfaction after microvascular depression and partial sensory rhizotomy for trigeminal neuralgia. Neurosurgery. 2005;56:1304-1311.

12. A Nanda, V Javalkar, S Zhang, O Ahmed. Long term efficacy and patient satisfaction of microvascular decompression and gamma knife radiosurgery for trigeminal neuralgia. Journal of Clinical Neuroscience 2015;22:818-822.

13. Linskey ME, Ratanatharathorn V, Peñagaricano J. A prospective cohort study of microvascular decompression and Gamma Knife surgery in patients with trigeminal. J Neurosurg. 2008;109 Suppl:160-172.

14. Zakrzewska JM, Thomas DG. Patient's assessment of outcome after three surgical procedures for the management of trigeminal neuralgia. Acta Neurochir (Wien). 1993;122:225-230.

15. Sindrup SH, Jensen TS.. Pharmacotherapy of trigeminal neuralgia. Clin J Pain 2002;18:22-27.

16. Wiffen PJ, McQuay HJ, Moore RA. Carbamazepine for acute and chronic pain. Cochrane

For peer review only - http://bmjopen.bmj.com/site/about/guidelines.xhtml

Database Syst Rev. 2005;(3):CD005451.

17. Zakrzewska JM, Patsalos PN. Long-term cohort study comparing medical (oxcarbazepine) and surgical management of intractable trigeminal neuralgia. Pain. 2002;95:259-66

18. Zakrzewska JM, Chaudhry Z, Nurmikko TJ, et al. Lamotrigine (Lamictal) in refractory trigeminal neuralgia: results from a double-blind placebo controlled crossover trial. Pain. 1997;73:223-230.

19. Court JE, Kase CS. Treatment of tic douloureux with a new anticonvulsant (clonazepam). J Neurol Neurosurg Psychiatry. 1976;39:297-299.

20. Yuan M, Zhou HY, Xiao ZL, et al. Efficacy and Safety of Gabapentin vs. Carbamazepine in the Treatment of Trigeminal Neuralgia: A Meta-Analysis. Pain Pract. 2016;16:1083-1091

21. Obermann M, Yoon MS, Sensen K, Maschke M, Diener HC, Katsarava Z. Efficacy of pregabalin in the treatment of trigeminal neuralgia. Cephalalgia. 2008;28:174-181.

22. Peiris JB, Perera GL, Devendra SV, Lionel ND. Sodium valproate in trigeminal neuralgia. Med J Aust. 1980;2:278.

23. Zakrzewska JM, Linskey ME. Trigeminal neuralgia. Clin Evid. 2014;10:1207

24. McQuay H, Carroll D, Jadad AR, et al. Anticonvulsant drugs for management of pain: a systematic review. BMJ. 1995;311:1047-1052.

25. Wiffen PJ, Rees J. Lamotrigine for acute and chronic pain. Cochrane Database Syst Rev. 2007;(2):CD006044.

26. Wiffen PJ, McQuay HJ, Rees J, Moore RA. Gabapentin for acute and chronic pain. Cochrane Database Syst Rev. 2005;(3):CD005452.

27. Wiffen PJ, Derry S, Moore RA, McQuay HJ. Carbamazepine for acute and chronic pain in adults. Cochrane Database Syst Rev. 2011;(1):CD005451.

28. Moore RA, Straube S, Wiffen PJ, Derry S, McQuay HJ. Pregabalin for acute and chronic pain in adults. Cochrane Database Syst Rev. 2009;(3):CD007076.

29. Caldwell DM, Ades AE, Higgins JP. Simultaneous comparison of multiple treatments: combining direct and indirect evidence. BMJ. 2005;331:897-900.

30. Cipriani A, Higgins JP, Geddes JR, Salanti G. Conceptual and technical challenges in network meta-analysis. Ann Intern Med. 2013;159:130-137.

31. Cipriani A, Furukawa TA, Salanti G, et al. Comparative efficacy and acceptability of 12 new-generation antidepressants: a multiple-treatments meta-analysis. Lancet. 2009;373:746-758.

32. Shamseer L, Moher D, Clarke M, et al. Preferred reporting items for systematic review and meta-analysis protocols (PRISMA-P) 2015: elaboration and explanation. BMJ. 2015;349:g7647.

33. Hutton B, Salanti G, Caldwell DM, et al. The PRISMA Extension Statement for Reporting of Systematic Reviews Incorporating Network Meta-analyses of Health Care Interventions: Checklist and Explanations. Ann Intern Med. 2015;162:777-784.

34. Merskey H, Bogduk N. Classification of chronic pain. Descriptions of chronic pain syndromes and definitions of pain terms, IASP Press, Seattle 1994, pp. 59-71.

For peer review only - http://bmjopen.bmj.com/site/about/guidelines.xhtml

For peer review only

35. Zakrzewska JM. Diagnosis and differential diagnosis of trigeminal neuralgia. Clin J Pain. 2002;18:14-21.

36. Dworkin RH, Turk DC, Farrar JT, et al. Core outcome measures for chronic pain clinical trials: IMMPACT recommendations. Pain. 2005;113:9-19.

37. Ware JE Jr, Sherbourne CD. The MOS 36-item short-form health survey (SF-36). I. Conceptual framework and item selection. Med Care. 1992;30:473-483.

38. Qin Z, Li B, Wu J, et al. Acupuncture for chronic diarrhea in adults: protocol for a systematic review. Medicine (Baltimore). 2017;94:e5952.

39. Higgins JPT, Altman DG, Sterne JAC (editors). Chapter 8: Assessing risk of bias in included studies. In: Higgins JPT, Green S(editors). Cochrane Handbook for Systematic Reviews of Intervention Version 5.1.0 [updated March 2011]. The Cochrane Collaboration, 2011. Available from www.cochrane-handbook.org.

40. Salanti G, Del Giovane C, Chaimani A, et al. Evaluating the quality of evidence from a network meta-analysis. PLoS ONE. 2014;9:e99682.

41. Furukawa TA, Cipriani A, Barbui C, Brambilla P, Watanabe N. Imputing response rates from means and standard deviations in meta-analyses. Int Clin Psychopharmacol. 2005;20:49-52.

42. Zhang J, Carlin B.P, Neaton J.D, et al. Network meta-analysis of randomized clinical trials: Reporting the proper summaries. Clinical Trials. 2014;11:246-262

43. Chaimani A, Higgins JPT, Mavridis D, Spyridonos P, Salanti G. Graphical tools for network meta-analysis in STATA. PLoS One. 2013;8:e76654.

44. Higgins JP, Thompson SG, Deeks JJ, Altman DG. Measuring inconsistency in meta-analyses. BMJ. 2003;327:557-560.

45. Baker SG, Kramer BS. The transitive fallacy for randomized trials: if A bests B and B bests C in separate trials, is A better than C? BMC Med Res Methodol. 2002;2:13.

46. White IR, Barrett JK, Jackson D, et al. Consistency and inconsistency in network meta-analysis: model estimation using multivariate meta-regression. Res Synth Methods. 2012;3:111-125

47. Higgins JPT, Jackson D, Barrett JL, et al. Consistency and inconsistency in network meta-analysis: concepts and models for multi-arm studies. Res Synth Methods. 2012;3:98-110

48. Dias S, Welton NJ, Caldwell DM, et al. Checking consistency in mixed treatment comparison meta-analysis. Stat Med. 2010;29:932-944

49. Lu G, Ades AE. Assessing evidence inconsistency in mixed treatment comparisions. J Am Stat Assoc. 2006;101:447-459.

50. Veroniki AA, Vasiliadis HS, Higgins JP, Salanti G. Evaluation of inconsistency in networks of interventions. Int J Epidemiol. 2013;42:332-345.

For peer review only

For peer review only - http://bmjopen.bmj.com/site/about/guidelines.xhtml

Figure legends of figure:

Figure 1. Network plot of all possible direct comparisons between the eligible interventions.

Figure 2. PRISMA flow chart.

For peer review only

For peer review only - http://bmjopen.bmj.com/site/about/guidelines.xhtml

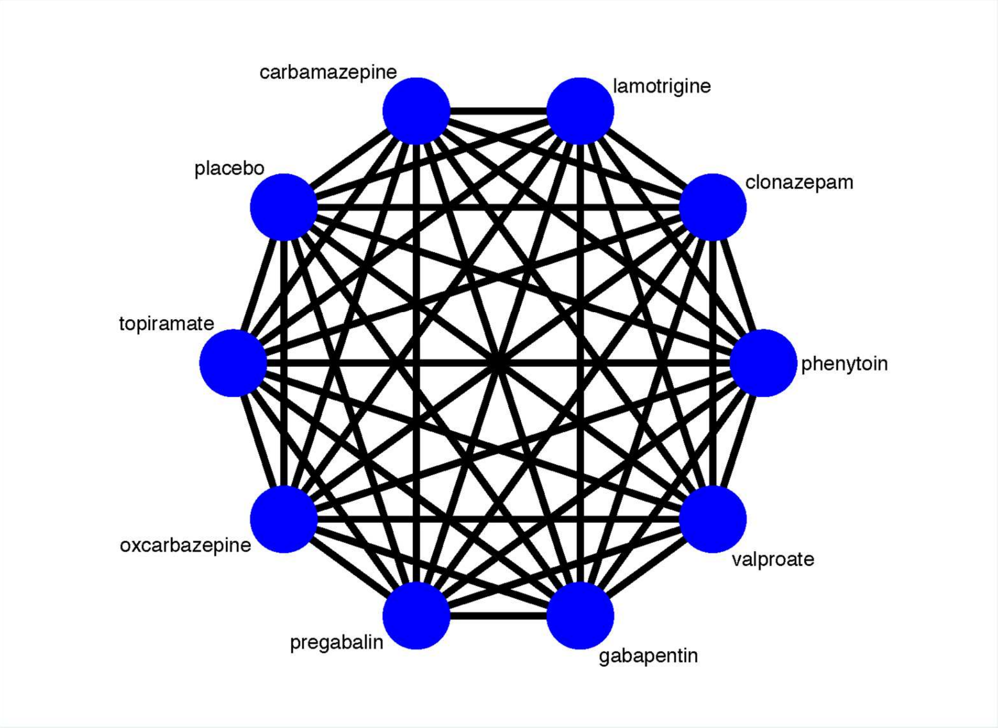

Network plot of all possible direct comparisons between the eligible interventions.

140x101mm (300 x 300 DPI)

For peer review only

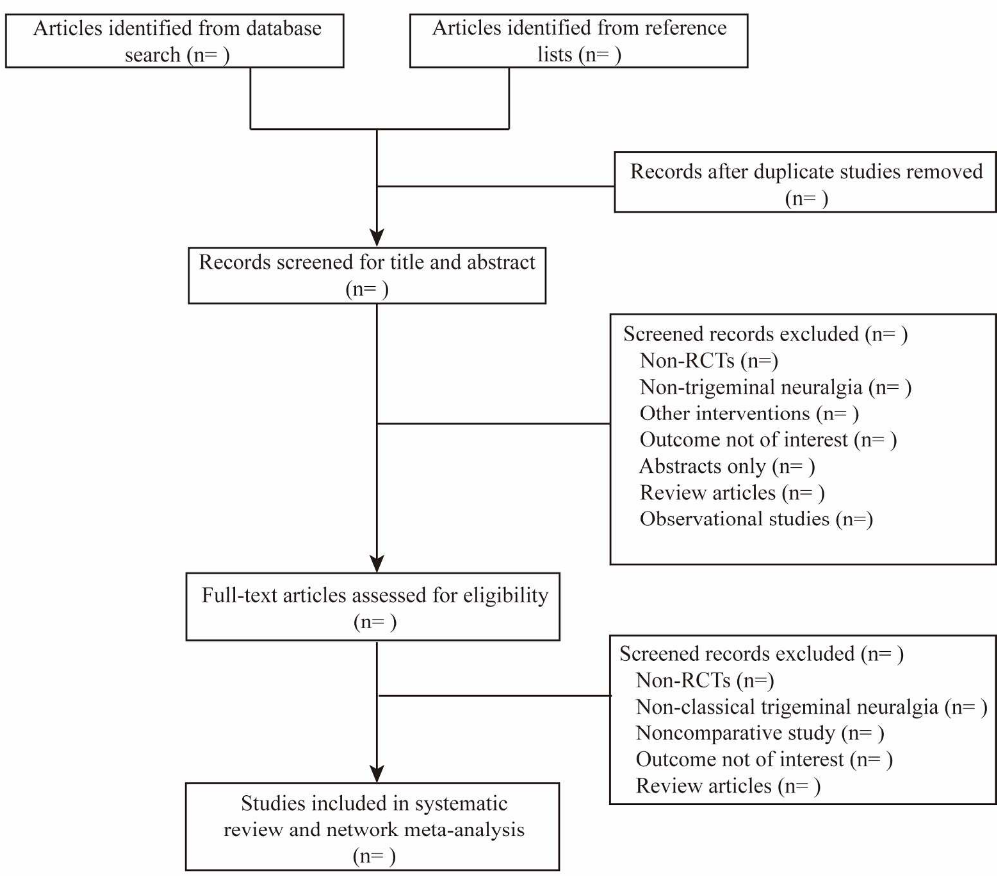

PRISMA flow chart

192x219mm (300 x 300 DPI)

PRISMA-P (Preferred Reporting Items for Systematic review and Meta-Analysis Protocols) 2015 checklist: recommended items to address in a systematic review protocol*

Section and topic	Item No	Checklist item
**ADMINISTRATIVE INFORMATION**		
Title:		
Identification	1a	Identify the report as a protocol of a systematic review Page 1
Update	1b	If the protocol is for an update of a previous systematic review, identify as such NA
Registration	2	If registered, provide the name of the registry (such as PROSPERO) and registration number Page 2
Authors:		
Contact	3a	Provide name, institutional affiliation, e-mail address of all protocol authors; provide physical mailing address of corresponding author Page 1
Contributions	3b	Describe contributions of protocol authors and identify the guarantor of the review Page 11
Amendments	4	If the protocol represents an amendment of a previously completed or published protocol, identify as such and list changes; otherwise, state plan for documenting important protocol amendments Page 11
Support:		
Sources	5a	Indicate sources of financial or other support for the review Page 11
Sponsor	5b	Provide name for the review funder and/or sponsor Page 11
Role of sponsor or funder	5c	Describe roles of funder(s), sponsor(s), and/or institution(s), if any, in developing the protocol Page 11
**INTRODUCTION**		
Rationale	6	Describe the rationale for the review in the context of what is already known Page 3
Objectives	7	Provide an explicit statement of the question(s) the review will address with reference to participants, interventions, comparators, and outcomes (PICO) Page 4
**METHODS**		
Eligibility criteria	8	Specify the study characteristics (such as PICO, study design, setting, time frame) and report characteristics (such as years considered, language, publication status) to be used as criteria for eligibility for the review Page 4
Information sources	9	Describe all intended information sources (such as electronic databases, contact with study authors, trial registers or other grey literature sources) with planned dates of coverage Page 5
Search strategy	10	Present draft of search strategy to be used for at least one electronic database, including planned limits, such that it could be repeated Page 6
Study records:		
Data management	11a	Describe the mechanism(s) that will be used to manage records and data throughout the review Page 6-7

For peer review only

Selection process	11b	State the process that will be used for selecting studies (such as two independent reviewers) through each phase of the review (that is, screening, eligibility and inclusion in meta-analysis) Page 6-7
Data collection process	11c	Describe planned method of extracting data from reports (such as piloting forms, done independently, in duplicate), any processes for obtaining and confirming data from investigators Page 6-7
Data items	12	List and define all variables for which data will be sought (such as PICO items, funding sources), any pre-planned data assumptions and simplifications Page 6-7
Outcomes and prioritization	13	List and define all outcomes for which data will be sought, including prioritization of main and additional outcomes, with rationale Page 5-6
Risk of bias in individual studies	14	Describe anticipated methods for assessing risk of bias of individual studies, including whether this will be done at the outcome or study level, or both; state how this information will be used in data synthesis Page 7
Data synthesis	15a	Describe criteria under which study data will be quantitatively synthesised Page 7-8
	15b	If data are appropriate for quantitative synthesis, describe planned summary measures, methods of handling data and methods of combining data from studies, including any planned exploration of consistency (such as I^2, Kendall's τ) Page 8
	15c	Describe any proposed additional analyses (such as sensitivity or subgroup analyses, meta-regression) Page 9-10
	15d	If quantitative synthesis is not appropriate, describe the type of summary planned Page 9-10
Meta-bias(es)	16	Specify any planned assessment of meta-bias(es) (such as publication bias across studies, selective reporting within studies) Page 7
Confidence in cumulative evidence	17	Describe how the strength of the body of evidence will be assessed (such as GRADE) Page 7

*** It is strongly recommended that this checklist be read in conjunction with the PRISMA-P Explanation and Elaboration (cite when available) for important clarification on the items. Amendments to a review protocol should be tracked and dated.** **The copyright for PRISMA-P (including checklist) is held by the PRISMA-P Group and is distributed under a Creative Commons Attribution Licence 4.0.**

From: Shamseer L, Moher D, Clarke M, Ghersi D, Liberati A, Petticrew M, Shekelle P, Stewart L, PRISMA-P Group. Preferred reporting items for systematic review and meta-analysis protocols (PRISMA-P) 2015: elaboration and explanation. BMJ. 2015 Jan 2;349(jan02 1):g7647.

For peer review only

PubMed

Patient

#1 "Trigeminal Neuralgia"[Mesh]

#2 Trigeminal Neuralgia*[Title/Abstract] OR Trifacial Neuralgia*[Title/Abstract] OR Fothergill Disease[Title/Abstract] OR Tic Douloureux[Title/Abstract] OR Epileptiform Neuralgia*[Title/Abstract] OR trigeminus neuralgia[Title/Abstract] OR prosopalgia[Title/Abstract] OR prosoponeuralgia[Title/Abstract] OR trigeminal nerve neuralgia[Title/Abstract] OR trigeminal nerve neuropathy[Title/Abstract] OR trigeminal neuropathy[Title/Abstract] OR trigeminus nerve neuralgia[Title/Abstract] OR trigeminus nerve neuropathy[Title/Abstract]

#3 #1 OR #2

RCT

#4 "Clinical Trials, Phase II as Topic"[Mesh] OR "Clinical Trials, Phase III as Topic"[Mesh] OR "Clinical Trials, Phase IV as Topic"[Mesh] OR "Controlled Clinical Trials as Topic"[Mesh] OR "Randomized Controlled Trials as Topic"[Mesh] OR "Intention to Treat Analysis"[Mesh] OR "Pragmatic Clinical Trials as Topic"[Mesh] OR "Clinical Trials, Phase II"[Publication Type] OR "Clinical Trials, Phase III"[Publication Type] OR "Clinical Trials, Phase IV"[Publication Type] OR "Controlled Clinical Trials"[Publication Type] OR "Randomized Controlled Trials"[Publication Type] OR "Pragmatic Clinical Trials as Topic"[Publication Type] OR "Single-Blind Method"[Mesh] OR "Double-Blind Method"[Mesh]

#5 random*[Title/Abstract] OR blind*[Title/Abstract] OR singleblind*[Title/Abstract] OR doubleblind*[Title/Abstract] OR trebleblind*[Title/Abstract] OR tripleblind*[Title/Abstract]

#6 #4 OR #5

#7 #3 AND #6

EMBASE.com

Patient

#1 'trigeminus neuralgia'/exp

#2 'trigeminal neuralgias':ab,ti OR 'trifacial neuralgias':ab,ti OR 'trigeminal neuralgia':ab,ti OR 'trifacial neuralgia':ab,ti OR 'fothergill disease':ab,ti OR 'tic douloureux':ab,ti OR 'epileptiform neuralgia':ab,ti OR 'epileptiform neuralgias':ab,ti OR 'trigeminus neuralgia':ab,ti OR prosopalgia:ab,ti OR prosoponeuralgia:ab,ti OR 'trigeminal nerve neuralgia':ab,ti OR 'trigeminal nerve neuropathy':ab,ti OR 'rigeminal neuropathy':ab,ti OR 'trigeminus nerve neuralgia':ab,ti OR 'trigeminus nerve neuropathy':ab,ti

#3 #1 OR #2

RCT

#4 'multicenter study (topic)'/exp OR 'phase 2 clinical trial (topic)'/exp OR 'phase 3 clinical trial

For peer review only

(topic)'/exp OR 'phase 4 clinical trial (topic)'/exp OR 'controlled clinical trial (topic)'/exp OR 'randomized controlled trial (topic)'/exp OR 'single blind procedure'/exp OR 'double blind procedure'/exp

#5 random*:ab,ti OR blind*:ab,ti OR singleblind*:ab,ti OR doubleblind*:ab,ti OR trebleblind*:ab,ti OR tripleblind*:ab,ti

#6 #4 OR #5

#7 #3 AND #6

Cochrane Library

#1 MeSH descriptor: [Trigeminal Neuralgia] explode all trees

#2 Trigeminal Neuralgia*:ti,ab,kw or Trifacial Neuralgia*:ti,ab,kw or Fothergill Disease:ti,ab,kw or Tic Douloureux:ti,ab,kw or Epileptiform Neuralgia*:ti,ab,kw or trigeminus neuralgia:ti,ab,kw or prosopalgia:ti,ab,kw or prosoponeuralgia:ti,ab,kw or trigeminal nerve neuralgia:ti,ab,kw or trigeminal nerve neuropathy:ti,ab,kw or trigeminal neuropathy:ti,ab,kw or trigeminus nerve neuralgia:ti,ab,kw or trigeminus nerve neuropathy:ti,ab,kw

#3 #1 or #2

For peer review only

Comparative Efficacy and Acceptability of Antiepileptic Drugs for Classical Trigeminal Neuralgia: A Bayesian Network Meta-Analysis Protocol

Journal:	*BMJ Open*
Manuscript ID	bmjopen-2017-017392.R2
Article Type:	Protocol
Date Submitted by the Author:	29-Aug-2017

Complete List of Authors:	Qin, Zongshi; Guang'anmen Hospital, China Academy of Chinese Medical Sciences, Department of Acupuncture and Neurology<br>Xie, Shang; Peking University School and Hospital of Stomatology, Department of Oral and Maxillofacial Surgery<br>Mao, Zhi; Chinese People's Liberation Army General Hospital, Department of Critical Care Medicine<br>Liu, Yan; China Academy of Chinese Medical Sciences, Data Centre of Traditional Chinese Medicine<br>WU, Jiani; Guang'anmen Hospital, China Academy of Chinese Medical Sciences, Department of Acupuncture and Neurology<br>Furukawa, Toshi; Kyoto University, Graduate School of Medicine and School of Public Health<br>Kwong, Joey; Taipei Medical University, Cochrane Taiwan; National Center for Child Health and Development, Department of Health Policy & Department of Clinical Epidemiology<br>Tian, Jinhui; Evidence based medicine center,<br>Liu, Zhishun; Guang'anmen Hospital, China Academy of Chinese Medical Sciences, Department of Acupuncture and Neurology

**Primary Subject Heading**:	Evidence based practice
Secondary Subject Heading:	Neurology
Keywords:	antiepileptic drugs, trigeminal neuralgia, network meta-analysis, systematic review, protocol

SCHOLARONE™ Manuscripts

Comparative Efficacy and Acceptability of Antiepileptic Drugs for Classical Trigeminal Neuralgia: A Bayesian Network Meta-Analysis Protocol

Zongshi Qin,[1] Shang Xie[2], Zhi Mao[3], Yan Liu[4], Jiani Wu[1], Toshi A Furukawa[5], Joey S.W. Kwong[6,7], Jinhui Tian[8], Zhishun Liu[1]

[1] Department of Acupuncture and Neurology, Guang'anmen Hospital, China Academy of Chinese Medical Sciences, Beijing, China

[2] Department of Oral and Maxillofacial Surgery, Peking University School and Hospital of Stomatology, Beijing, China

[3] Department of Critical Care Medicine, Chinese People's Liberation Army General Hospital, Beijing, China

[4] Data Centre of Traditional Chinese Medicine, China Academy of Chinese Medical Sciences, Beijing, China

[5] Department of Health Promotion and Human Behavior, Kyoto University Graduate School of Medicine/School of Public Health, Kyoto, Japan

[6] Cochrane Taiwan, Taipei Medical University, Taipei, Taiwan

[7] Department of Health Policy & Department of Clinical Epidemiology, National Center for Child Health and Development, Tokyo, Japan

[8] Evidence-based Medicine Center, Lanzhou University, Lanzhou, China

ZQ, SX and ZM contributed equally to this work.

Correspondence to: Jinhui Tian, email: tianjh@lzu.edu.cn; Zhishun Liu, email: liuzhishun@aliyun.com

Keywords: antiepileptic drugs; trigeminal neuralgia; network meta-analysis; systematic review; protocol

For peer review only - http://bmjopen.bmj.com/site/about/guidelines.xhtml

ABSTRACT

Introduction Trigeminal neuralgia (TN) affects 4 to 28.9/100,000 people worldwide, and antiepileptic drugs such as carbamazepine and oxcarbazepine are the first-line treatment options. However, the efficacy and safety of other antiepileptic drugs remain unclear due to insufficient direct comparisons.

Objective To compare the efficacy and acceptability of all currently available antiepileptic agents for the treatment of patients with classical TN.

Methods We will search the PubMed, EMBASE, Cochrane Library, and Web of Science databases for unpublished or undergoing research listed in registry platforms. We will include all randomized controlled trials comparing two different antiepileptic drugs or one antiepileptic drug with placebo in patients with classic TN. The primary outcomes will be the proportion of responders and the number of subjects who drop out during the treatment. The secondary outcomes include the two primary outcomes but set in the follow-up period, changes in the self-reporting assessment scale for neuralgia, and quality of life assessment. In terms of network meta-analysis, we will fit our model in a Bayesian framework using WinBUGS (Medical Research Council's Biostatistics Unit, Cambridge, UK). To confirm the results, we will also conduct analyses using Stata (Version 13.0; Stata Corporation, College Station, Texas, USA) and compare the differences between the two platforms.

Ethics and dissemination This protocol will not disseminate any private patient data. The results of this review will be disseminated through peer-reviewed publication.

Protocol registration for this systematic review (registration number): PROSPERO (CRD: 42016048640).

Strengths and limitations of this study

- To the best of the authors' knowledge, this study will be the first network meta-analysis that assess the comparative efficacy and acceptability of all the available antiepileptic drugs for the classical trigeminal neuralgia.
- This study will be performed by Bayesian framework, which enables us to estimate the probability for each intervention to be the best for each outcome.
- Owing to the language barrier, the amount of included trials might be potentially limited.

For peer review only - http://bmjopen.bmj.com/site/about/guidelines.xhtml

Introduction

Classical trigeminal neuralgia (TN), a chronic pain disorder described as one of the most severe pains one can suffer, is characterized by paroxysms of unilateral, electric shock-like, and severe pain along the trigeminal nerve divisions.[1,2] It affects lifestyle because it can be triggered by common activities such as eating, talking, shaving and brushing teeth. The wind, chewing and talking also aggravate the condition in many patients.[2] It is estimated that approximately 4 to 28.9 per 100,000 people worldwide suffer from TN, and the number affected tends to be higher among women at all ages and even increases with age.[3,4]

At present, the cause of TN remains unclear.[5,6] One hypotheses is that the trigeminal nerve becomes compressed at the root entry zone by cerebral vessels.[7] Owing to the contradictory etiology and poorly understood pathophysiological mechanisms underlying TN, a variety of therapeutic and surgical approaches have been developed to alleviate the associated pain and improve the quality of life in patients with classical TN.[8-10] Although many patients have obtained excellent outcomes from surgery, many others do not experience any pain relief.[11,12] Furthermore, the currently available surgical procedures are associated with various complications, particularly sensory loss in the trigeminal nerve territory, anesthesia dolorosa and, rarely, ipsilateral hearing loss, depending on the technique.[13,14]

As such, pharmacological measures to improve clinical outcomes are needed. The most commonly used option is antiepileptic drugs, with phenytoin being the first drug used for classical TN with positive effect.[15] Carbamazepine can reduce both the frequency and intensity of painful paroxysms and was first introduced by the US Food and Drug Administration (FDA); however, its efficacy is compromised by poor tolerability.[16] Oxcarbazepine, a derivative of carbamazepine, is often used as an initial treatment for classical TN and has more favorable properties than carbamazepine related to its increased efficacy in epilepsy, greater tolerability and decreased potential for drug interactions.[17] Lamotrigine has also been reported as an effective add-on therapy,[18] whereas little evidence supports that other antiepileptic drugs such as clonazepam, gabapentin, pregabalin and valproate have a beneficial effect.[19-22] However, many of the studies are old with limited methodology, and were assessed as low GRADE scores.[23]

To date, several systematic reviews have investigated the comparative efficacy and safety of antiepileptic drugs.[20,24-28] However, previous systematic reviews have only considered pair-wise

For peer review only

evidence from head-to-head comparisons and have thus failed to assess the comparative efficacy and acceptability of all available antiepileptic drugs. Thus, it is difficult to determine the best treatments for relieving pain with minimal adverse effects. In the present study, we choose a group of 9 antiepileptic drugs looking at the drugs which were licensed for neuralgia in many countries and which were frequently used in clinical practice. We will apply network meta-analysis to integrate direct and indirect comparisons,[29,30] which could be used not only to strengthen inferences concerning the efficacy and acceptability of treatments but also to rank the efficacy and acceptability of antiepileptic drugs accordingly.[31]

The objectives of this systematic review and network meta-analysis are 1) to compare all currently available antiepileptic drugs in terms of efficacy and acceptability in classical TN treatment and 2) to determine which drug achieves the best balance between efficacy and adverse effects. The results of this study will augment findings based on current pair-wise meta-analyses and are expected to provide important information to support clinical practice and health policy decisions.

METHODS

This protocol will be conducted in accordance with the reporting guidance provided in the Preferred Reporting Items for Systematic Reviews and Meta-Analysis Protocols (PRISMA-P) statement and Checklist of Items to Include When Reporting a Systematic Review Involving a Network Meta-analysis.[32,33] The protocol is registered in PROSPERO (CRD: 42016048640). This study will not involve any private patient data, ethical approval was waived. (supplemental file 1 represents the PRISMA-P checklist)

Eligibility criteria

Study types

We will include randomized controlled trials (RCTs) comparing one antiepileptic drug with another antiepileptic drug as monotherapy or placebo for the treatment of TN. Quasi-randomized controlled trails allocating participants according to birth date or the consequences of enrollment will be excluded. The minimum duration for RCT inclusion was set at 4 weeks. Trials with more than a two-arm design will be considered only if the available data meet the criteria for an intervention. For trials with a crossover design, data will only be extracted from the first

For peer review only - http://bmjopen.bmj.com/site/about/guidelines.xhtml

randomization period.

Participant characteristics

Only trials that enrolled participants with a diagnosis of classical TN according to standardized criteria such as the classification of the International Headache Society (IHS) of International Classification of Headache Disorders will be sought.[1,34] For studies using other extensive criteria for the diagnosis of classical TN, detailed diagnostic criteria must be reported (such as history or characteristics that have been confirmed by CT or MRI).[35] Studies examining symptomatic TN patients will not be included. Participants with comorbid conditions such as anxiety, depression, epilepsy or other medical conditions will also not be eligible for inclusion. No limitations will be imposed for age, sex, or nationality.

Intervention types

We plan to include the following antiepileptic drugs: carbamazepine, lamotrigine, clonazepam, phenytoin, valproate, gabapentin, pregabalin, oxcarbazepine and topiramate. In addition to these antiepileptic drugs, we will also obtain information about interventions of interest from either pair-wise RCTs or placebo-controlled trails, as some RCTs design a placebo-controlled arm as the comparator. Figure 1 illustrates the network plot of all possible direct comparisons between the eligible interventions.

Outcome measures

Studies reporting one of the following will be included.

Primary outcome

The primary objective of this review is to assess the efficacy and acceptability of antiepileptic drugs for classical TN; therefore, the following two outcomes will be used as the primary outcome.

1. The proportion of responders to a self-reporting assessment scale for neuralgia. A responder was defined as a subject who obtained a ≥ 50% pain reduction score from baseline to endpoint (4–12 weeks) or a subject who obtained a pain reducing score of no less than the minimal clinically important difference (MCID). Pain scores will be extracted based on the visual analogue score

For peer review only

(VAS), numerical rating score (NRS), or any other validated scale for the assessment of overall TN symptoms when available.[36]

2. Treatment acceptability is defined as the proportion of patients who have intervention related adverse events during the 4 to 12 weeks.

Secondary outcomes

1. The proportion of responders with ≥ 50% pain reduction on a self-reporting assessment scale for neuralgia from baseline to endpoint after follow-up.

2. The change in pain symptoms of TN from baseline to endpoint (4–12 weeks) measured based on the VAS, NRS, or any other validated scale for the assessment of overall TN symptoms when available.

3. The change in pain symptoms of TN from baseline to endpoint after follow-up.

4. The quality of life based on measurement with a validated scale, such as the Short Form 36 Health Survey questionnaire (SF-36).[37]

Search strategy

We will identify RCTs through a comprehensive, systematic literature search primarily utilizing the PubMed, EMBASE, Cochrane Library, and Web of Science databases. As publication bias caused by insufficient unpublished data can significantly bias the comparative efficacy results of network meta-analysis and modify rankings, we will also perform searches for unpublished or ongoing trials using System for information on Grey Literature in Europe (SIGLE) as well as other registry platforms, such as Clinicaltrials.gov and the International Clinical Trials Registry Platform. Prior to completing this review, we will perform an additional search of each database and registration platform to guarantee that the most recent studies are included. We will use medical subject headings and text words related to 'trigeminal neuralgia' and 'randomized controlled trial' for the literature search. In addition, the reference lists of previous systematic reviews will be examined to ensure the quantity and accuracy of the included studies. The search strategy will be developed by JT and ZL, we anticipate that the aforementioned databases will be searched from their inception to 30th September, 2017. (supplemental file 2 represents the search strategies for PubMed, EMBASE and Cochrane Library)

For peer review only - http://bmjopen.bmj.com/site/about/guidelines.xhtml

For peer review only

Data collection process

Two authors (SX and ZM) will scan the titles and abstracts of the trials after duplicated records have been excluded using EndNote X7 (Thomson Reuters, New York, NY). The scanning will be performed using EndNote, and all trials will be allocated to the following five groups: inclusion group, non-patient group, intervention group, outcome group, and awaiting group. A prior data collection process will be conducted using an electro-table created with Excel software, which has been used in our previous study.[38] The table will consist of four sheets, including general information (author list, publication year, and journal), characteristics of included trials (diagnostic criteria, age range, study drugs, and dose range), the risk of bias assessed using the Cochrane risk of bias tool, and outcome data extraction (number of participants who responded to treatment and the number who dropped out during the treatment). All original data will be submitted as an attachment. A flow chart illustrating this design is presented in Figure 2.

Quality assessment

Two authors (JW and YL) will use the Cochrane risk of bias tool to assess the risk of bias of eligible studies, covering randomization, concealment allocation, blinding and other biases.[39] As inadequate concealment could potentially fail the randomization test, two independent review authors will pay particular attention to the adequacy of random allocation concealment and blinding. The other sources of bias will be assessed while considering sample size calculation method, diagnostic criteria, reporting of withdrawals and follow-up. Two authors (JK and JT) will assess the quality of evidence using Grading of Recommendations Assessment, Development and Evaluation (GRADE) framework, covering study limitations, inconsistency, indirectness, imprecision and publication bias.[40] The methods for rating the quality of direct comparisons are the same to the methods used in traditional meta-analysis, and following steps will be used in the whole assessment procedure: 1) presenting direct and indirect effect estimates; 2) rating the quality of direct and indirect estimates; 3) presenting the results of network meta-analysis; 4) rating the quality of network meta-analysis effect estimates.

Dealing with missing data

To obtain missing data, we will initially contact the senior or corresponding author. If no one

For peer review only - http://bmjopen.bmj.com/site/about/guidelines.xhtml

responds, we will estimate the missing data as follows. For studies failing to report the number of responding patients after treatment, instead of providing the mean and standard deviation, we will calculate the number of responding patients employing a validated imputation method.[41] In addition, we will also estimate missing data from graphs when possible. For trials that cannot be extracted or estimated, the available data will be excluded, and the reason for exclusion will be reported.

Statistical analysis

The method used for data synthesis will be based on mixed treatment meta-analysis. To examine comparisons, we will use Stata (Version 13.0; Stata Corporation, College Station, Texas, USA) to synthesize data and will present the comparison results if the included studies are sufficient for each pair-wise comparison. We will use a random effects model to combine the data, and the outcomes of continuous and binary variables will be presented as standardized mean differences (SMDs) and odds ratios (ORs) with 95% confidential intervals (CIs). For indirect comparisons, network meta-analysis will be developed in a Bayesian framework using Markov chain Monte Carlo simulation methods in WinBUGS (Medical Research Council's Biostatistics Unit, Cambridge, UK) with a Chaimani model.[42] The results of network meta-analysis will use the arm-based parameterization for random effects model.[43] This will enable us to estimate the best probability for each intervention for each positive outcome, given the results of the multiple-treatment meta-analysis. At least one network focusing on the response rate for pain relief will be constructed, in which a statistically significant difference defined as the null value will not be included in the 95% CI. The Markov chains will be utilized for 50,000 simultaneous iterations based on the data and the description of the proposed distributions for relevant parameters, and the first 10,000 iterations will be discarded to avoid potential impact on the arbitrary value. For continuous outcomes and binary outcomes, the OR and SMD will be presented with the 95% credible interval (CrI). In this process, the Brooks-Gelman-Rubin method will be used to assess the convergence between direct and indirect variances. According to the theory of Brooks and Gelman, if a potential scale reduction factor (PSRF) is less than 1.2, then an approximate convergence has occurred. The PSRF results will be presented graphically using a Brooks-Gelman-Rubin diagnosis plot, if needed. To describe relationships among different

For peer review only - http://bmjopen.bmj.com/site/about/guidelines.xhtml

treatments, a network plot will be created to show direct comparisons between arms based on different outcomes. To confirm the results, we will also conduct the same network meta-analysis using the network package of Stata (http://www.mrc-bsu.cam.ac.uk/IW_Stata/), and the outcome will be compared to that produced using WinBUGS. In addition, the effectiveness of each treatment among all available treatments will be ranked by calculating the OR in order, and plots of the surfaces under the cumulative ranking curves (SUCRAs) will be generated to rank the various treatments for each outcome using Stata software.[44] We will also present a cluster rank table to synthesize the efficacy and acceptability of each drug (using two primary outcomes). The table will consist of two triangles: the upper right triangle will illustrate the acceptability, and the lower left triangle will illustrate the efficacy.[31]

Assessment of heterogeneity

Heterogeneity, which plays a pivotal role in both standard meta-analysis and network meta-analysis, refers to the degree of disagreement between study-specific treatment effects and constitutes the basis of inconsistency. To test the heterogeneity of each pair-wise comparison, we will use the I^2 statistic.[45]

Assessment of transitivity and similarity

In addition to the heterogeneity assessment using the I^2 statistic, the assumption of transitivity and similarity based on clinical and methodological characteristics will be assessed. It should be noted that it is difficult to identify these effect modifiers using statistical analysis. We will assume that intervention effects are transitive in this network meta-analysis because we will only focus on antiepileptic drugs, and we will investigate similarity based on clinical characteristics, such as antiepileptic drug dose, period of treatment, and severity of pain symptoms at baseline, as well as according to methodological characteristics such as study quality.[46] All these effect modifiers will be judged and reported before the network meta-analysis is conducted.

Assessment of inconsistency

The evaluation and explanation of inconsistency is another basic objective of network meta-analysis. In this context, inconsistency refers to the degree of difference between direct and indirect comparisons and can be evaluated only when a loop exists in the evidence network. This

For peer review only - http://bmjopen.bmj.com/site/about/guidelines.xhtml

means that inconsistency assessment using a design-by-treatment interaction model cannot be conducted if the structure of this network is a "star network" (i.e., all interventions have a single mutual comparator, such as a placebo).[47,48] For such cases, we will test inconsistency using a node-splitting model.[49]

To identify inconsistency among the included trials of the network, we will use Stata, performing the Z test to compare direct and indirect summary effects in specific loops.[50] If there is no inconsistency between loops or designs, we will use a consistency model to calculate the data. For cases of significant incoherence, we will initially look for data extraction errors in loops that present inconsistency and in comparisons with large heterogeneity.[51] After the data have been scrutinized, we will investigate possible sources of inconsistency within clinical and methodological variables suspected of being potential sources of either heterogeneity or incoherence in each comparison-specific group of trials. If an important inconsistency cannot be explained, we will consider avoiding synthesis of the related network.

Additional analyses

To ensure the quality of this review, studies not reporting blinding will be excluded prior to data synthesis because blinding plays a vital important role in the randomized controlled trial. We will assess heterogeneity quantitatively using the I^2 statistic, and if an I^2 value is greater than 50%, then we will explore the source of heterogeneity. We will initially perform sensitivity analysis by excluding trials rated as having a high risk of bias. Additionally, meta-regression or subgroup analysis will be used to explore possible sources of heterogeneity if the number of included trials is sufficient. For network meta-regression, we will use a random effects network meta-regression model to examine potential factors.

Discussion

To the best of our knowledge, no network meta-analyses comparing the use of antiepileptic drugs for treatment of classical TN have been conducted to date. Previous systematic reviews have compared only a single drug to other types of drug or therapy.[20,24-28] This makes it difficult to obtain a clear understanding of the effectiveness of the various different conservative treatments for this disorder. Network meta-analysis can be used to perform indirect comparisons and allows

For peer review only - http://bmjopen.bmj.com/site/about/guidelines.xhtml

parameters for direct and indirect comparisons to be synthesized. To ensure the quantity and quality of the potentially included RCTs, we will perform an extensive literature search and predefine rigorous inclusion criteria. Besides, we will assess the quality of evidence with the GRADE framework. Although a ranking of the included interventions will be generated, with the exception of findings, the quality of evidence should also be considered. We hope that the results of this review help clinicians make more accurate treatment decisions and promote additional research into conservative treatments for classical TN.

For peer review only

For peer review only - http://bmjopen.bmj.com/site/about/guidelines.xhtml

Amendments

If it is necessary to update this protocol, we will update this protocol in the future. We will submit the original protocol, final protocol and summary of changes as a supplement.

Authors' contributions

ZQ, SX, and ZM conceived of the study. JT and SX developed the search strategies. ZQ, JT and SX wrote the first draft. TAF and ZL revised the draft. SX and ZM will independently screen potential studies and extract data from the included studies. JW, JK, JT and YL will assess the risk of bias and summarize the evidence. ZM, SX, JK and ZL will address the missing data, if any. ZQ and JT will perform the statistical analysis. ZL and JT will arbitrate in cases of disagreement and ensure the absence of errors. All authors approve the publication of this protocol.

Ethics and dissemination

This protocol will not disseminate any private patient data. The results of this review will be disseminated through peer-reviewed publication.

Competing interests: None.

Funding: None.

For peer review only

For peer review only - http://bmjopen.bmj.com/site/about/guidelines.xhtml

Reference

1. Headache Classification Committee of the International Headache Society (IHS). The International Classification of Headache Disorders, 3rd edition (beta version). Cephalalgia. 2013;33:629-808.

2. Zakrzewska JM, Linskey ME. Trigeminal neuralgia. BMJ. 2015;350:h1238

3. Meller D, Obermann M, Yoon MS, Poitz F, Hansen N, Slomke MA. Prevalence of trigeminal neuralgia and persistent idiopathic facial pain: a population-based study. Cephalalgia. 2011;31:1542-1548.

4. MacDonald BK, Cockerell OC, Sander JW, Shorvon SD. The incidence and lifetime prevalence of neurological disorders in a prospective community-based study in the UK. Brain. 2000;123:665-676.

5. Devor M, Amir R, Rappaport ZH. Pathophysiology of trigeminal neuralgia: the ignition hypothesis. Clin J Pain. 2002;18:4-13.

6. Danielle DD, Hodaie M, Davis KD. Structural magnetic resonance imaging can identify trigeminal system abnormalities in classical trigeminal neuralgia. Front Neuroanat. 2016;10:95.

7. Katusic S, Beard CM, Bergstralh E, Kurland LT. Incidence and clinical features of trigeminal neuralgia, Rochester, Minnesota, 1945-1984. Ann Neurol. 1990;27:89-95.

8. Gronseth G, Cruccu G, Alksne J, et al. Practice parameter: the diagnostic evaluation and treatment of trigeminal neuralgia (an evidence-based review): report of the Quality Standards Subcommittee of the American Academy of Neurology and the European Federation of Neurological Societies. Neurology. 2008;71:1183-1190.

9. Campbell FG, Graham JG, Zilkha KJ. Clinical trial of carbazepine (tegretol) in trigeminal neuralgia. J Neurol Neurosurg Psychiatry. 1966;29:265-267.

10. Fromm GH, Terrence CF, Chattha AS. Baclofen in the treatment of trigeminal neuralgia: double-blind study and long-term follow-up. Ann Neurol. 1984;15:240-244.

11. Zakrzewska JM, Lopez BC, Kim SE, Coakham HB. Patient reports of satisfaction after microvascular depression and partial sensory rhizotomy for trigeminal neuralgia. Neurosurgery. 2005;56:1304-1311.

12. A Nanda, V Javalkar, S Zhang, O Ahmed. Long term efficacy and patient satisfaction of microvascular decompression and gamma knife radiosurgery for trigeminal neuralgia. Journal of Clinical Neuroscience 2015;22:818-822.

13. Linskey ME, Ratanatharathorn V, Peñagaricano J. A prospective cohort study of microvascular decompression and Gamma Knife surgery in patients with trigeminal. J Neurosurg. 2008;109 Suppl:160-172.

14. Zakrzewska JM, Thomas DG. Patient's assessment of outcome after three surgical procedures for the management of trigeminal neuralgia. Acta Neurochir (Wien). 1993;122:225-230.

15. Sindrup SH, Jensen TS.. Pharmacotherapy of trigeminal neuralgia. Clin J Pain 2002;18:22-27.

16. Wiffen PJ, McQuay HJ, Moore RA. Carbamazepine for acute and chronic pain. Cochrane

For peer review only - http://bmjopen.bmj.com/site/about/guidelines.xhtml

Database Syst Rev. 2005;(3):CD005451.

17. Zakrzewska JM, Patsalos PN. Long-term cohort study comparing medical (oxcarbazepine) and surgical management of intractable trigeminal neuralgia. Pain. 2002;95:259-66

18. Zakrzewska JM, Chaudhry Z, Nurmikko TJ, et al. Lamotrigine (Lamictal) in refractory trigeminal neuralgia: results from a double-blind placebo controlled crossover trial. Pain. 1997;73:223-230.

19. Court JE, Kase CS. Treatment of tic douloureux with a new anticonvulsant (clonazepam). J Neurol Neurosurg Psychiatry. 1976;39:297-299.

20. Yuan M, Zhou HY, Xiao ZL, et al. Efficacy and Safety of Gabapentin vs. Carbamazepine in the Treatment of Trigeminal Neuralgia: A Meta-Analysis. Pain Pract. 2016;16:1083-1091

21. Obermann M, Yoon MS, Sensen K, Maschke M, Diener HC, Katsarava Z. Efficacy of pregabalin in the treatment of trigeminal neuralgia. Cephalalgia. 2008;28:174-181.

22. Peiris JB, Perera GL, Devendra SV, Lionel ND. Sodium valproate in trigeminal neuralgia. Med J Aust. 1980;2:278.

23. Zakrzewska JM, Linskey ME. Trigeminal neuralgia. Clin Evid. 2014;10:1207

24. McQuay H, Carroll D, Jadad AR, et al. Anticonvulsant drugs for management of pain: a systematic review. BMJ. 1995;311:1047-1052.

25. Wiffen PJ, Rees J. Lamotrigine for acute and chronic pain. Cochrane Database Syst Rev. 2007;(2):CD006044.

26. Wiffen PJ, McQuay HJ, Rees J, Moore RA. Gabapentin for acute and chronic pain. Cochrane Database Syst Rev. 2005;(3):CD005452.

27. Wiffen PJ, Derry S, Moore RA, McQuay HJ. Carbamazepine for acute and chronic pain in adults. Cochrane Database Syst Rev. 2011;(1):CD005451.

28. Moore RA, Straube S, Wiffen PJ, Derry S, McQuay HJ. Pregabalin for acute and chronic pain in adults. Cochrane Database Syst Rev. 2009;(3):CD007076.

29. Caldwell DM, Ades AE, Higgins JP. Simultaneous comparison of multiple treatments: combining direct and indirect evidence. BMJ. 2005;331:897-900.

30. Cipriani A, Higgins JP, Geddes JR, Salanti G. Conceptual and technical challenges in network meta-analysis. Ann Intern Med. 2013;159:130-137.

31. Cipriani A, Furukawa TA, Salanti G, et al. Comparative efficacy and acceptability of 12 new-generation antidepressants: a multiple-treatments meta-analysis. Lancet. 2009;373:746-758.

32. Shamseer L, Moher D, Clarke M, et al. Preferred reporting items for systematic review and meta-analysis protocols (PRISMA-P) 2015: elaboration and explanation. BMJ. 2015;349:g7647.

33. Hutton B, Salanti G, Caldwell DM, et al. The PRISMA Extension Statement for Reporting of Systematic Reviews Incorporating Network Meta-analyses of Health Care Interventions: Checklist and Explanations. Ann Intern Med. 2015;162:777-784.

34. Merskey H, Bogduk N. Classification of chronic pain. Descriptions of chronic pain syndromes and definitions of pain terms, IASP Press, Seattle 1994, pp. 59-71.

For peer review only - http://bmjopen.bmj.com/site/about/guidelines.xhtml

35. Zakrzewska JM. Diagnosis and differential diagnosis of trigeminal neuralgia. Clin J Pain. 2002;18:14-21.

36. Dworkin RH, Turk DC, Farrar JT, et al. Core outcome measures for chronic pain clinical trials: IMMPACT recommendations. Pain. 2005;113:9-19.

37. Ware JE Jr, Sherbourne CD. The MOS 36-item short-form health survey (SF-36). I. Conceptual framework and item selection. Med Care. 1992;30:473-483.

38. Qin Z, Li B, Wu J, et al. Acupuncture for chronic diarrhea in adults: protocol for a systematic review. Medicine (Baltimore). 2017;94:e5952.

39. Higgins JPT, Altman DG, Sterne JAC (editors). Chapter 8: Assessing risk of bias in included studies. In: Higgins JPT, Green S(editors). Cochrane Handbook for Systematic Reviews of Intervention Version 5.1.0 [updated March 2011]. The Cochrane Collaboration, 2011. Available from www.cochrane-handbook.org.

40. Salanti G, Del Giovane C, Chaimani A, et al. Evaluating the quality of evidence from a network meta-analysis. PLoS ONE. 2014;9:e99682.

41. Furukawa TA, Cipriani A, Barbui C, Brambilla P, Watanabe N. Imputing response rates from means and standard deviations in meta-analyses. Int Clin Psychopharmacol. 2005;20:49-52.

42. Lu G, Ades AE. Combination of direct and indirect evidence in mixed treatment comparisons. Stat Med. 2004;23:3105–3124

43. Zhang J, Carlin B.P, Neaton J.D, et al. Network meta-analysis of randomized clinical trials: Reporting the proper summaries. Clinical Trials. 2014;11:246-262

44. Chaimani A, Higgins JPT, Mavridis D, Spyridonos P, Salanti G. Graphical tools for network meta-analysis in STATA. PLoS One. 2013;8:e76654.

45. Higgins JP, Thompson SG, Deeks JJ, Altman DG. Measuring inconsistency in meta-analyses. BMJ. 2003;327:557-560.

46. Baker SG, Kramer BS. The transitive fallacy for randomized trials: if A bests B and B bests C in separate trials, is A better than C? BMC Med Res Methodol. 2002;2:13.

47. White IR, Barrett JK, Jackson D, et al. Consistency and inconsistency in network meta-analysis: model estimation using multivariate meta-regression. Res Synth Methods. 2012;3:111-125

48. Higgins JPT, Jackson D, Barrett JL, et al. Consistency and inconsistency in network meta-analysis: concepts and models for multi-arm studies. Res Synth Methods. 2012;3:98-110

49. Dias S, Welton NJ, Caldwell DM, et al. Checking consistency in mixed treatment comparison meta-analysis. Stat Med. 2010;29:932-944

50. Lu G, Ades AE. Assessing evidence inconsistency in mixed treatment comparisions. J Am Stat Assoc. 2006;101:447-459.

51. Veroniki AA, Vasiliadis HS, Higgins JP, Salanti G. Evaluation of inconsistency in networks of interventions. Int J Epidemiol. 2013;42:332-345.

For peer review only - http://bmjopen.bmj.com/site/about/guidelines.xhtml

Figure legends of figure:

Figure 1. Network plot of all possible direct comparisons between the eligible interventions.

Figure 2. PRISMA flow chart.

For peer review only

For peer review only - http://bmjopen.bmj.com/site/about/guidelines.xhtml

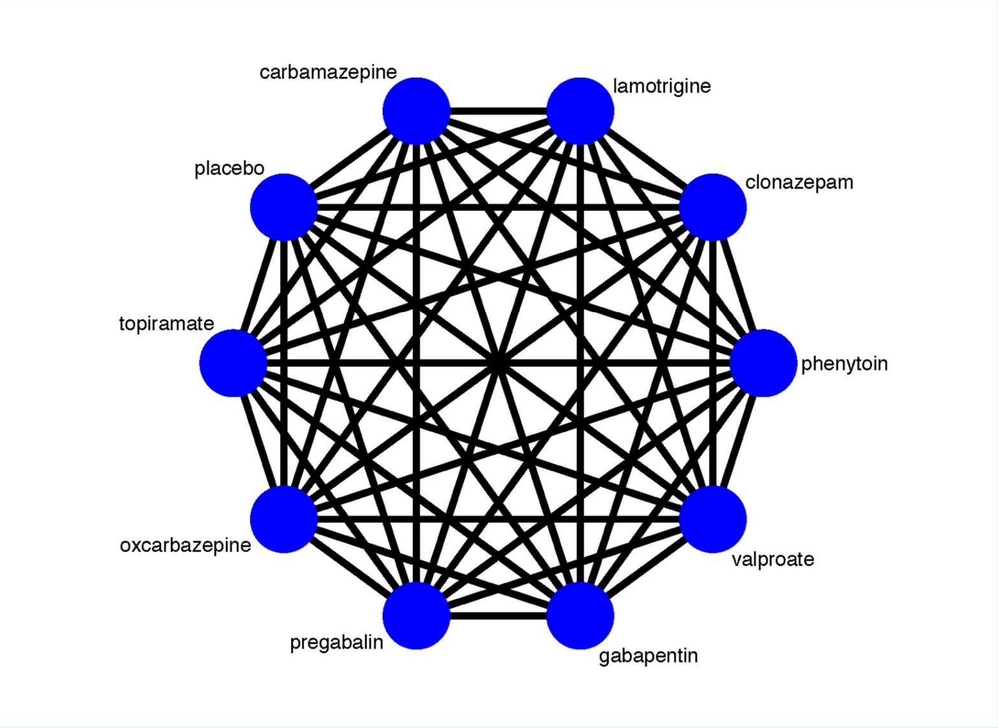

Network plot of all possible direct comparisons between the eligible interventions.

140x101mm (300 x 300 DPI)

For peer review only

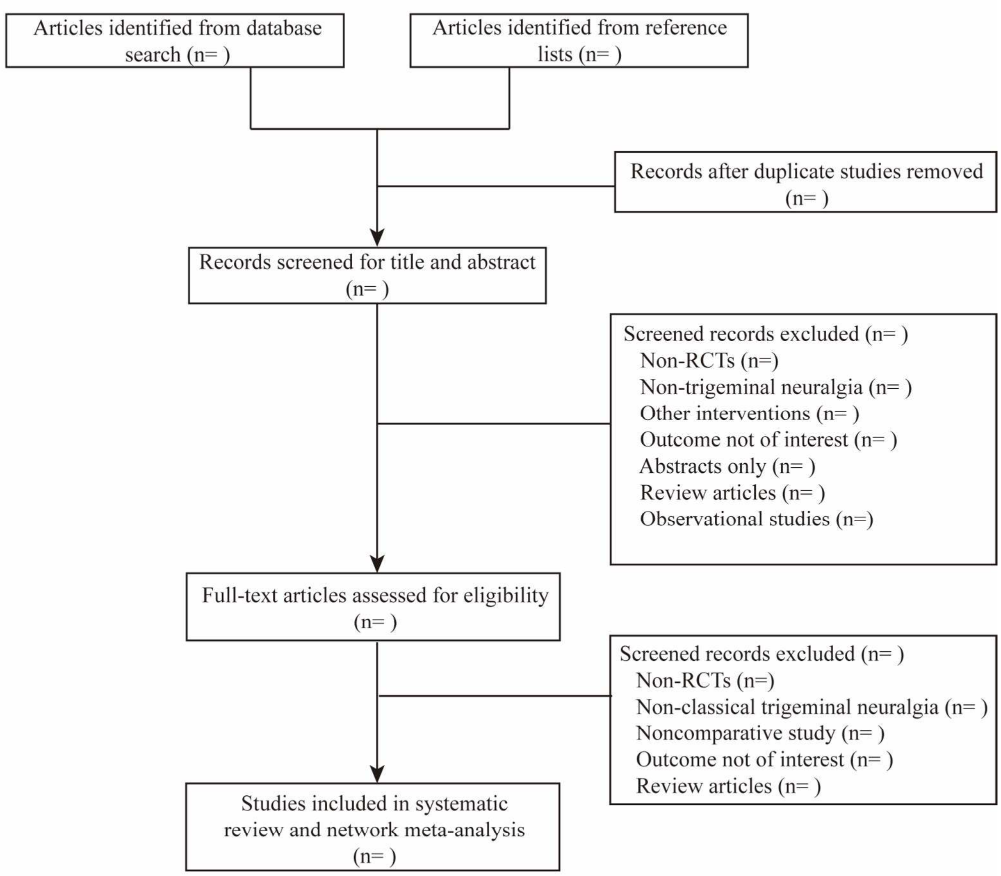

PRISMA flow chart

192x219mm (300 x 300 DPI)

PRISMA-P (Preferred Reporting Items for Systematic review and Meta-Analysis Protocols) 2015 checklist: recommended items to address in a systematic review protocol*

Section and topic	Item No	Checklist item
**ADMINISTRATIVE INFORMATION**		
Title:		
Identification	1a	Identify the report as a protocol of a systematic review Page 1
Update	1b	If the protocol is for an update of a previous systematic review, identify as such NA
Registration	2	If registered, provide the name of the registry (such as PROSPERO) and registration number Page 2
Authors:		
Contact	3a	Provide name, institutional affiliation, e-mail address of all protocol authors; provide physical mailing address of corresponding author Page 1
Contributions	3b	Describe contributions of protocol authors and identify the guarantor of the review Page 11
Amendments	4	If the protocol represents an amendment of a previously completed or published protocol, identify as such and list changes; otherwise, state plan for documenting important protocol amendments Page 11
Support:		
Sources	5a	Indicate sources of financial or other support for the review Page 11
Sponsor	5b	Provide name for the review funder and/or sponsor Page 11
Role of sponsor or funder	5c	Describe roles of funder(s), sponsor(s), and/or institution(s), if any, in developing the protocol Page 11
**INTRODUCTION**		
Rationale	6	Describe the rationale for the review in the context of what is already known Page 3
Objectives	7	Provide an explicit statement of the question(s) the review will address with reference to participants, interventions, comparators, and outcomes (PICO) Page 4
**METHODS**		
Eligibility criteria	8	Specify the study characteristics (such as PICO, study design, setting, time frame) and report characteristics (such as years considered, language, publication status) to be used as criteria for eligibility for the review Page 4
Information sources	9	Describe all intended information sources (such as electronic databases, contact with study authors, trial registers or other grey literature sources) with planned dates of coverage Page 5
Search strategy	10	Present draft of search strategy to be used for at least one electronic database, including planned limits, such that it could be repeated Page 6
Study records:		
Data management	11a	Describe the mechanism(s) that will be used to manage records and data throughout the review Page 6-7

For peer review only

Selection process	11b	State the process that will be used for selecting studies (such as two independent reviewers) through each phase of the review (that is, screening, eligibility and inclusion in meta-analysis) Page 6-7
Data collection process	11c	Describe planned method of extracting data from reports (such as piloting forms, done independently, in duplicate), any processes for obtaining and confirming data from investigators Page 6-7
Data items	12	List and define all variables for which data will be sought (such as PICO items, funding sources), any pre-planned data assumptions and simplifications Page 6-7
Outcomes and prioritization	13	List and define all outcomes for which data will be sought, including prioritization of main and additional outcomes, with rationale Page 5-6
Risk of bias in individual studies	14	Describe anticipated methods for assessing risk of bias of individual studies, including whether this will be done at the outcome or study level, or both; state how this information will be used in data synthesis Page 7
Data synthesis	15a	Describe criteria under which study data will be quantitatively synthesised Page 7-8
	15b	If data are appropriate for quantitative synthesis, describe planned summary measures, methods of handling data and methods of combining data from studies, including any planned exploration of consistency (such as I^2, Kendall's τ) Page 8
	15c	Describe any proposed additional analyses (such as sensitivity or subgroup analyses, meta-regression) Page 9-10
	15d	If quantitative synthesis is not appropriate, describe the type of summary planned Page 9-10
Meta-bias(es)	16	Specify any planned assessment of meta-bias(es) (such as publication bias across studies, selective reporting within studies) Page 7
Confidence in cumulative evidence	17	Describe how the strength of the body of evidence will be assessed (such as GRADE) Page 7

*** It is strongly recommended that this checklist be read in conjunction with the PRISMA-P Explanation and Elaboration (cite when available) for important clarification on the items. Amendments to a review protocol should be tracked and dated. The copyright for PRISMA-P (including checklist) is held by the PRISMA-P Group and is distributed under a Creative Commons Attribution Licence 4.0.**

From: Shamseer L, Moher D, Clarke M, Ghersi D, Liberati A, Petticrew M, Shekelle P, Stewart L, PRISMA-P Group. Preferred reporting items for systematic review and meta-analysis protocols (PRISMA-P) 2015: elaboration and explanation. BMJ. 2015 Jan 2;349(jan02 1):g7647.

For peer review only

PubMed

Patient

#1 "Trigeminal Neuralgia"[Mesh]

#2 Trigeminal Neuralgia*[Title/Abstract] OR Trifacial Neuralgia*[Title/Abstract] OR Fothergill Disease[Title/Abstract] OR Tic Douloureux[Title/Abstract] OR Epileptiform Neuralgia*[Title/Abstract] OR trigeminus neuralgia[Title/Abstract] OR prosopalgia[Title/Abstract] OR prosoponeuralgia[Title/Abstract] OR trigeminal nerve neuralgia[Title/Abstract] OR trigeminal nerve neuropathy[Title/Abstract] OR trigeminal neuropathy[Title/Abstract] OR trigeminus nerve neuralgia[Title/Abstract] OR trigeminus nerve neuropathy[Title/Abstract]

#3 #1 OR #2

RCT

#4 "Clinical Trials, Phase II as Topic"[Mesh] OR "Clinical Trials, Phase III as Topic"[Mesh] OR "Clinical Trials, Phase IV as Topic"[Mesh] OR "Controlled Clinical Trials as Topic"[Mesh] OR "Randomized Controlled Trials as Topic"[Mesh] OR "Intention to Treat Analysis"[Mesh] OR "Pragmatic Clinical Trials as Topic"[Mesh] OR "Clinical Trials, Phase II"[Publication Type] OR "Clinical Trials, Phase III"[Publication Type] OR "Clinical Trials, Phase IV"[Publication Type] OR "Controlled Clinical Trials"[Publication Type] OR "Randomized Controlled Trials"[Publication Type] OR "Pragmatic Clinical Trials as Topic"[Publication Type] OR "Single-Blind Method"[Mesh] OR "Double-Blind Method"[Mesh]

#5 random*[Title/Abstract] OR blind*[Title/Abstract] OR singleblind*[Title/Abstract] OR doubleblind*[Title/Abstract] OR trebleblind*[Title/Abstract] OR tripleblind*[Title/Abstract]

#6 #4 OR #5

#7 #3 AND #6

EMBASE.com

Patient

#1 'trigeminus neuralgia'/exp

#2 'trigeminal neuralgias':ab,ti OR 'trifacial neuralgias':ab,ti OR 'trigeminal neuralgia':ab,ti OR 'trifacial neuralgia':ab,ti OR 'fothergill disease':ab,ti OR 'tic douloureux':ab,ti OR 'epileptiform neuralgia':ab,ti OR 'epileptiform neuralgias':ab,ti OR 'trigeminus neuralgia':ab,ti OR prosopalgia:ab,ti OR prosoponeuralgia:ab,ti OR 'trigeminal nerve neuralgia':ab,ti OR 'trigeminal nerve neuropathy':ab,ti OR 'rigeminal neuropathy':ab,ti OR 'trigeminus nerve neuralgia':ab,ti OR 'trigeminus nerve neuropathy':ab,ti

#3 #1 OR #2

RCT

#4 'multicenter study (topic)'/exp OR 'phase 2 clinical trial (topic)'/exp OR 'phase 3 clinical trial

For peer review only

(topic)'/exp OR 'phase 4 clinical trial (topic)'/exp OR 'controlled clinical trial (topic)'/exp OR 'randomized controlled trial (topic)'/exp OR 'single blind procedure'/exp OR 'double blind procedure'/exp

#5 random*:ab,ti OR blind*:ab,ti OR singleblind*:ab,ti OR doubleblind*:ab,ti OR trebleblind*:ab,ti OR tripleblind*:ab,ti

#6 #4 OR #5

#7 #3 AND #6

Cochrane Library

#1 MeSH descriptor: [Trigeminal Neuralgia] explode all trees

#2 Trigeminal Neuralgia*:ti,ab,kw or Trifacial Neuralgia*:ti,ab,kw or Fothergill Disease:ti,ab,kw or Tic Douloureux:ti,ab,kw or Epileptiform Neuralgia*:ti,ab,kw or trigeminus neuralgia:ti,ab,kw or prosopalgia:ti,ab,kw or prosoponeuralgia:ti,ab,kw or trigeminal nerve neuralgia:ti,ab,kw or trigeminal nerve neuropathy:ti,ab,kw or trigeminal neuropathy:ti,ab,kw or trigeminus nerve neuralgia:ti,ab,kw or trigeminus nerve neuropathy:ti,ab,kw

#3 #1 or #2

For peer review only

Comparative Efficacy and Acceptability of Antiepileptic Drugs for Classical Trigeminal Neuralgia: A Bayesian Network Meta-Analysis Protocol

Journal:	*BMJ Open*
Manuscript ID	bmjopen-2017-017392.R3
Article Type:	Protocol
Date Submitted by the Author:	24-Oct-2017
Complete List of Authors:	Qin, Zongshi; Guang'anmen Hospital, China Academy of Chinese Medical Sciences, Department of Acupuncture and Neurology<br>Xie, Shang; Peking University School and Hospital of Stomatology, Department of Oral and Maxillofacial Surgery<br>Mao, Zhi; Chinese People's Liberation Army General Hospital, Department of Critical Care Medicine<br>Liu, Yan; China Academy of Chinese Medical Sciences, Data Centre of Traditional Chinese Medicine<br>WU, Jiani; Guang'anmen Hospital, China Academy of Chinese Medical Sciences, Department of Acupuncture and Neurology<br>Furukawa, Toshi; Kyoto University, Graduate School of Medicine and School of Public Health<br>Kwong, Joey; Taipei Medical University, Cochrane Taiwan; National Center for Child Health and Development, Department of Health Policy & Department of Clinical Epidemiology<br>Tian, Jinhui; Evidence based medicine center,<br>Liu, Zhishun; Guang'anmen Hospital, China Academy of Chinese Medical Sciences, Department of Acupuncture and Neurology
**Primary Subject Heading**:	Evidence based practice
Secondary Subject Heading:	Neurology
Keywords:	antiepileptic drugs, trigeminal neuralgia, network meta-analysis, systematic review, protocol

SCHOLARONE™
Manuscripts

Comparative Efficacy and Acceptability of Antiepileptic Drugs for Classical Trigeminal Neuralgia: A Bayesian Network Meta-Analysis Protocol

Zongshi Qin,[1] Shang Xie[2], Zhi Mao[3], Yan Liu[4], Jiani Wu[1], Toshi A Furukawa[5], Joey S.W. Kwong[6,7], Jinhui Tian[8], Zhishun Liu[1]

[1] Department of Acupuncture and Neurology, Guang'anmen Hospital, China Academy of Chinese Medical Sciences, Beijing, China

[2] Department of Oral and Maxillofacial Surgery, Peking University School and Hospital of Stomatology, Beijing, China

[3] Department of Critical Care Medicine, Chinese People's Liberation Army General Hospital, Beijing, China

[4] Data Centre of Traditional Chinese Medicine, China Academy of Chinese Medical Sciences, Beijing, China

[5] Department of Health Promotion and Human Behavior, Kyoto University Graduate School of Medicine/School of Public Health, Kyoto, Japan

[6] Cochrane Taiwan, Taipei Medical University, Taipei, Taiwan

[7] Department of Health Policy & Department of Clinical Epidemiology, National Center for Child Health and Development, Tokyo, Japan

[8] Evidence-based Medicine Center, Lanzhou University, Lanzhou, China

ZQ, SX and ZM contributed equally to this work.

Correspondence to: Zhishun Liu, email: liuzhishun@aliyun.com

Keywords: antiepileptic drugs; trigeminal neuralgia; network meta-analysis; systematic review; protocol

For peer review only - http://bmjopen.bmj.com/site/about/guidelines.xhtml

ABSTRACT

Introduction Trigeminal neuralgia (TN) affects 4 to 28.9/100,000 people worldwide, and antiepileptic drugs such as carbamazepine and oxcarbazepine are the first-line treatment options. However, the efficacy and safety of other antiepileptic drugs remain unclear due to insufficient direct comparisons.

Objective To compare the efficacy and acceptability of all currently available antiepileptic agents for the treatment of patients with classical TN.

Methods We will search the PubMed, EMBASE, Cochrane Library, and Web of Science databases for unpublished or undergoing research listed in registry platforms. We will include all randomized controlled trials comparing two different antiepileptic drugs or one antiepileptic drug with placebo in patients with classic TN. The primary outcomes will be the proportion of responders and the number of subjects who drop out during the treatment. The secondary outcomes include the two primary outcomes but set in the follow-up period, changes in the self-reporting assessment scale for neuralgia, and quality of life assessment. In terms of network meta-analysis, we will fit our model in a Bayesian framework using JAGS and pcnetmeta package of R project.

Ethics and dissemination This protocol will not disseminate any private patient data. The results of this review will be disseminated through peer-reviewed publication.

Protocol registration for this systematic review (registration number): PROSPERO (CRD: 42016048640).

Strengths and limitations of this study

- To the best of the authors' knowledge, this study will be the first network meta-analysis that assess the comparative efficacy and acceptability of all the available antiepileptic drugs for the classical trigeminal neuralgia.
- This study will be performed by Bayesian framework, which enables us to estimate the probability for each intervention to be the best for each outcome.
- Owing to the language barrier, the amount of included trials might be potentially limited.

For peer review only - http://bmjopen.bmj.com/site/about/guidelines.xhtml

Introduction

Classical trigeminal neuralgia (TN), a chronic pain disorder described as one of the most severe pains one can suffer, is characterized by paroxysms of unilateral, electric shock-like, and severe pain along the trigeminal nerve divisions.[1,2] It affects lifestyle because it can be triggered by common activities such as eating, talking, shaving and brushing teeth. The wind, chewing and talking also aggravate the condition in many patients.[2] It is estimated that approximately 4 to 28.9 per 100,000 people worldwide suffer from TN, and the number affected tends to be higher among women at all ages and even increases with age.[3,4]

At present, the cause of TN remains unclear.[5,6] One hypotheses is that the trigeminal nerve becomes compressed at the root entry zone by cerebral vessels.[7] Owing to the contradictory etiology and poorly understood pathophysiological mechanisms underlying TN, a variety of therapeutic and surgical approaches have been developed to alleviate the associated pain and improve the quality of life in patients with classical TN.[8-10] Although many patients have obtained excellent outcomes from surgery, many others do not experience any pain relief.[11,12] Furthermore, the currently available surgical procedures are associated with various complications, particularly sensory loss in the trigeminal nerve territory, anesthesia dolorosa and, rarely, ipsilateral hearing loss, depending on the technique.[13,14]

As such, pharmacological measures to improve clinical outcomes are needed. The most commonly used option is antiepileptic drugs, with phenytoin being the first drug used for classical TN with positive effect.[15] Carbamazepine can reduce both the frequency and intensity of painful paroxysms and was first introduced by the US Food and Drug Administration (FDA); however, its efficacy is compromised by poor tolerability.[16] Oxcarbazepine, a derivative of carbamazepine, is often used as an initial treatment for classical TN and has more favorable properties than carbamazepine related to its increased efficacy in epilepsy, greater tolerability and decreased potential for drug interactions.[17] Lamotrigine has also been reported as an effective add-on therapy,[18] whereas little evidence supports that other antiepileptic drugs such as clonazepam, gabapentin, pregabalin and valproate have a beneficial effect.[19-22] However, many of the studies are old with limited methodology, and were assessed as low GRADE scores.[23]

To date, several systematic reviews have investigated the comparative efficacy and safety of antiepileptic drugs.[20,24-28] However, previous systematic reviews have only considered pair-wise

For peer review only - http://bmjopen.bmj.com/site/about/guidelines.xhtml

evidence from head-to-head comparisons and have thus failed to assess the comparative efficacy and acceptability of all available antiepileptic drugs. Thus, it is difficult to determine the best treatments for relieving pain with minimal adverse effects. In the present study, we choose a group of 9 antiepileptic drugs looking at the drugs which were licensed for neuralgia in many countries and which were frequently used in clinical practice. We will apply network meta-analysis to integrate direct and indirect comparisons,[29,30] which could be used not only to strengthen inferences concerning the efficacy and acceptability of treatments but also to rank the efficacy and acceptability of antiepileptic drugs accordingly.[31]

The objectives of this systematic review and network meta-analysis are 1) to compare all currently available antiepileptic drugs in terms of efficacy and acceptability in classical TN treatment and 2) to determine which drug achieves the best balance between efficacy and adverse effects. The results of this study will augment findings based on current pair-wise meta-analyses and are expected to provide important information to support clinical practice and health policy decisions.

METHODS

This protocol will be conducted in accordance with the reporting guidance provided in the Preferred Reporting Items for Systematic Reviews and Meta-Analysis Protocols (PRISMA-P) statement and Checklist of Items to Include When Reporting a Systematic Review Involving a Network Meta-analysis.[32,33] The protocol is registered in PROSPERO (CRD: 42016048640). This study will not involve any private patient data, ethical approval was waived. (supplemental file 1 represents the PRISMA-P checklist)

Eligibility criteria

Study types

We will include randomized controlled trials (RCTs) comparing one antiepileptic drug with another antiepileptic drug as monotherapy or placebo for the treatment of TN. Quasi-randomized controlled trails allocating participants according to birth date or the consequences of enrollment will be excluded. The minimum duration for RCT inclusion was set at 4 weeks. Trials with more than a two-arm design will be considered only if the available data meet the criteria for an intervention. For trials with a crossover design, data will only be extracted from the first

For peer review only - http://bmjopen.bmj.com/site/about/guidelines.xhtml

randomization period.

Participant characteristics

Only trials that enrolled participants with a diagnosis of classical TN according to standardized criteria such as the classification of the International Headache Society (IHS) of International Classification of Headache Disorders will be sought.[1,34] For studies using other extensive criteria for the diagnosis of classical TN, detailed diagnostic criteria must be reported (such as history or characteristics that have been confirmed by CT or MRI).[35] Studies examining symptomatic TN patients will not be included. Participants with comorbid conditions such as anxiety, depression, epilepsy or other medical conditions will also not be eligible for inclusion. No limitations will be imposed for age, sex, or nationality.

Intervention types

We plan to include the following antiepileptic drugs: carbamazepine, lamotrigine, clonazepam, phenytoin, valproate, gabapentin, pregabalin, oxcarbazepine and topiramate. In addition to these antiepileptic drugs, we will also obtain information about interventions of interest from either pair-wise RCTs or placebo-controlled trails, as some RCTs design a placebo-controlled arm as the comparator. Figure 1 illustrates the network plot of all possible direct comparisons between the eligible interventions.

Outcome measures

Studies reporting one of the following will be included.

Primary outcome

The primary objective of this review is to assess the efficacy and acceptability of antiepileptic drugs for classical TN; therefore, the following two outcomes will be used as the primary outcome.

1. The proportion of responders to a self-reporting assessment scale for neuralgia. A responder was defined as a subject who obtained a ≥50% pain reduction score from baseline to endpoint (4–12 weeks) or a subject who obtained a pain reducing score of no less than the minimal clinically important difference (MCID). Pain scores will be extracted based on the visual analogue score

For peer review only - http://bmjopen.bmj.com/site/about/guidelines.xhtml

(VAS), numerical rating score (NRS), or any other validated scale for the assessment of overall TN symptoms when available.[36]

2. Treatment acceptability is defined as the proportion of patients who have intervention related adverse events during the 4 to 12 weeks.

Secondary outcomes

1. The proportion of responders with ≥50% pain reduction on a self-reporting assessment scale for neuralgia from baseline to endpoint after follow-up.

2. The change in pain symptoms of TN from baseline to endpoint (4–12 weeks) measured based on the VAS, NRS, or any other validated scale for the assessment of overall TN symptoms when available.

3. The change in pain symptoms of TN from baseline to endpoint after follow-up.

4. The quality of life based on measurement with a validated scale, such as the Short Form 36 Health Survey questionnaire (SF-36).[37]

Search strategy

We will identify RCTs through a comprehensive, systematic literature search primarily utilizing the PubMed, EMBASE, Cochrane Library, and Web of Science databases. As publication bias caused by insufficient unpublished data can significantly bias the comparative efficacy results of network meta-analysis and modify rankings, we will also perform searches for unpublished or ongoing trials using System for information on Grey Literature in Europe (SIGLE) as well as other registry platforms, such as Clinicaltrials.gov and the International Clinical Trials Registry Platform. Prior to completing this review, we will perform an additional search of each database and registration platform to guarantee that the most recent studies are included. We will use medical subject headings and text words related to ‘trigeminal neuralgia’ and ‘randomized controlled trial’ for the literature search. In addition, the reference lists of previous systematic reviews will be examined to ensure the quantity and accuracy of the included studies. The search strategy will be developed by JT and ZL, we anticipate that the aforementioned databases will be searched from their inception to 30th September, 2017. (supplemental file 2 represents the search strategies for PubMed, EMBASE and Cochrane Library)

For peer review only

Data collection process

Two authors (SX and ZM) will scan the titles and abstracts of the trials after duplicated records have been excluded using EndNote X7 (Thomson Reuters, New York, NY). The scanning will be performed using EndNote, and all trials will be allocated to the following five groups: inclusion group, non-patient group, intervention group, outcome group, and awaiting group. A prior data collection process will be conducted using an electro-table created with Excel software, which has been used in our previous study.[38] The table will consist of four sheets, including general information (author list, publication year, and journal), characteristics of included trials (diagnostic criteria, age range, study drugs, and dose range), the risk of bias assessed using the Cochrane risk of bias tool, and outcome data extraction (number of participants who responded to treatment and the number who dropped out during the treatment). All original data will be submitted as an attachment. A flow chart illustrating this design is presented in Figure 2.

Quality assessment

Two authors (JW and YL) will use the Cochrane risk of bias tool to assess the risk of bias of eligible studies, covering randomization, concealment allocation, blinding and other biases.[39] As inadequate concealment could potentially fail the randomization test, two independent review authors will pay particular attention to the adequacy of random allocation concealment and blinding. The other sources of bias will be assessed while considering sample size calculation method, diagnostic criteria, reporting of withdrawals and follow-up. Two authors (JK and JT) will assess the quality of evidence using Grading of Recommendations Assessment, Development and Evaluation (GRADE) framework, covering study limitations, inconsistency, indirectness, imprecision and publication bias.[40] The methods for rating the quality of direct comparisons are the same to the methods used in traditional meta-analysis, and following steps will be used in the whole assessment procedure: 1) presenting direct and indirect effect estimates; 2) rating the quality of direct and indirect estimates; 3) presenting the results of network meta-analysis; 4) rating the quality of network meta-analysis effect estimates.

Dealing with missing data

To obtain missing data, we will initially contact the senior or corresponding author. If no one

For peer review only - http://bmjopen.bmj.com/site/about/guidelines.xhtml

responds, we will estimate the missing data as follows. For studies failing to report the number of responding patients after treatment, instead of providing the mean and standard deviation, we will calculate the number of responding patients employing a validated imputation method.[41] In addition, we will also estimate missing data from graphs when possible. For trials that cannot be extracted or estimated, the available data will be excluded, and the reason for exclusion will be reported.

Statistical analysis

The method used for data synthesis will be based on mixed treatment meta-analysis. To examine comparisons, we will use Stata (13.0; Stata Corporation, College Station, Texas, USA) to synthesize data and will present the comparison results if the included studies are sufficient for each pair-wise comparison. We will use a random effects model to combine the data, and the outcomes of continuous and binary variables will be presented as standardized mean differences (SMDs) and odds ratios (ORs) with 95% confidential intervals (CIs). For indirect comparisons, we will perform arm-based network meta-analysis for all treatments using a random effects model with a Bayesian framework using the pcnetmeta package of R project, which could conduct calculation by calling JAGS software.[42-44] This will enable us to estimate the best probability for each intervention for each positive outcome, given the results of the multiple-treatment meta-analysis. At least one network focusing on the response rate for pain relief will be constructed, in which a statistically significant difference defined as the null value will not be included in the 95% CI. All model will be utilized for 50,000 simultaneous iterations based on the data and the description of the proposed distributions for relevant parameters, and the first 10,000 iterations will be discarded to avoid potential impact on the arbitrary value. For continuous outcomes and binary outcomes, the OR and SMD will be presented with the 95% credible interval (CrI). To describe relationships among different treatments, a network plot will be created to show direct comparisons between arms based on different outcomes.[42] In addition, the effectiveness of each treatment among all available treatments will be ranked by calculating the OR in order, and plots of the treatment rank probabilities will be generated to rank the various treatments for each outcome using the functions in package pcnetmeta.[42 43] We will also present a cluster rank table to synthesize the efficacy and acceptability of each drug (using two primary outcomes). The table

For peer review only - http://bmjopen.bmj.com/site/about/guidelines.xhtml

will consist of two triangles: the upper right triangle will illustrate the acceptability, and the lower left triangle will illustrate the efficacy.[31] For pair-wise meta-analyses we will use Stata 13.0. For network meta-analyses we will use JAGS and R project.

Assessment of heterogeneity

Heterogeneity, which plays a pivotal role in both standard meta-analysis and network meta-analysis, refers to the degree of disagreement between study-specific treatment effects and constitutes the basis of inconsistency. To test the heterogeneity of each pair-wise comparison, we will use the I^2 statistic.[45]

Assessment of transitivity and similarity

In addition to the heterogeneity assessment using the I^2 statistic, the assumption of transitivity and similarity based on clinical and methodological characteristics will be assessed. It should be noted that it is difficult to identify these effect modifiers using statistical analysis. We will assume that intervention effects are transitive in this network meta-analysis because we will only focus on antiepileptic drugs, and we will investigate similarity based on clinical characteristics, such as antiepileptic drug dose, period of treatment, and severity of pain symptoms at baseline, as well as according to methodological characteristics such as study quality.[46] All these effect modifiers will be judged and reported before the network meta-analysis is conducted.

Assessment of inconsistency

The evaluation and explanation of inconsistency is another basic objective of network meta-analysis. In this context, inconsistency refers to the degree of difference between direct and indirect comparisons and can be evaluated only when a loop exists in the evidence network. This means that inconsistency assessment using a design-by-treatment interaction model cannot be conducted if the structure of this network is a “star network” (i.e., all interventions have a single mutual comparator, such as a placebo).[47,48] For such cases, we will test inconsistency using a node-splitting model.[49]

To identify inconsistency among the included trials of the network, we will use Stata, performing the Z test to compare direct and indirect summary effects in specific loops.[50] If there is no inconsistency between loops or designs, we will use a consistency model to calculate the data. For

For peer review only - http://bmjopen.bmj.com/site/about/guidelines.xhtml

cases of significant incoherence, we will initially look for data extraction errors in loops that present inconsistency and in comparisons with large heterogeneity.[51] After the data have been scrutinized, we will investigate possible sources of inconsistency within clinical and methodological variables suspected of being potential sources of either heterogeneity or incoherence in each comparison-specific group of trials. If an important inconsistency cannot be explained, we will consider avoiding synthesis of the related network.

Additional analyses

To ensure the quality of this review, studies not reporting blinding will be excluded prior to data synthesis because blinding plays a vital important role in the randomized controlled trial. We will assess heterogeneity quantitatively using the I^2 statistic, and if an I^2 value is greater than 50%, then we will explore the source of heterogeneity. We will initially perform sensitivity analysis by excluding trials rated as having a high risk of bias. Additionally, meta-regression or subgroup analysis will be used to explore possible sources of heterogeneity if the number of included trials is sufficient. For network meta-regression, we will use a random effects network meta-regression model to examine potential factors.

Discussion

To the best of our knowledge, no network meta-analyses comparing the use of antiepileptic drugs for treatment of classical TN have been conducted to date. Previous systematic reviews have compared only a single drug to other types of drug or therapy.[20,24-28] This makes it difficult to obtain a clear understanding of the effectiveness of the various different conservative treatments for this disorder. Network meta-analysis can be used to perform indirect comparisons and allows parameters for direct and indirect comparisons to be synthesized. To ensure the quantity and quality of the potentially included RCTs, we will perform an extensive literature search and predefine rigorous inclusion criteria. Besides, we will assess the quality of evidence with the GRADE framework. Although a ranking of the included interventions will be generated, with the exception of findings, the quality of evidence should also be considered. We hope that the results of this review help clinicians make more accurate treatment decisions and promote additional research into conservative treatments for classical TN.

For peer review only - http://bmjopen.bmj.com/site/about/guidelines.xhtml

For peer review only - http://bmjopen.bmj.com/site/about/guidelines.xhtml

Amendments

If it is necessary to update this protocol, we will update this protocol in the future. We will submit the original protocol, final protocol and summary of changes as a supplement.

Authors' contributions

ZQ, SX, and ZM conceived of the study. JT and SX developed the search strategies. ZQ, JT and SX wrote the first draft. TAF and ZL revised the draft. SX and ZM will independently screen potential studies and extract data from the included studies. JW, JK, JT and YL will assess the risk of bias and summarize the evidence. ZM, SX, JK and ZL will address the missing data, if any. ZQ and JT will perform the statistical analysis. ZL and JT will arbitrate in cases of disagreement and ensure the absence of errors. All authors approve the publication of this protocol.

Ethics and dissemination

This protocol will not disseminate any private patient data. The results of this review will be disseminated through peer-reviewed publication.

Competing interests: None.

Funding: None.

For peer review only

For peer review only - http://bmjopen.bmj.com/site/about/guidelines.xhtml

Reference

1. Headache Classification Committee of the International Headache Society (IHS). The International Classification of Headache Disorders, 3rd edition (beta version). Cephalalgia. 2013;33:629-808.

2. Zakrzewska JM, Linskey ME. Trigeminal neuralgia. BMJ. 2015;350:h1238

3. Meller D, Obermann M, Yoon MS, Poitz F, Hansen N, Slomke MA. Prevalence of trigeminal neuralgia and persistent idiopathic facial pain: a population-based study. Cephalalgia. 2011;31:1542-1548.

4. MacDonald BK, Cockerell OC, Sander JW, Shorvon SD. The incidence and lifetime prevalence of neurological disorders in a prospective community-based study in the UK. Brain. 2000;123:665-676.

5. Devor M, Amir R, Rappaport ZH. Pathophysiology of trigeminal neuralgia: the ignition hypothesis. Clin J Pain. 2002;18:4-13.

6. Danielle DD, Hodaie M, Davis KD. Structural magnetic resonance imaging can identify trigeminal system abnormalities in classical trigeminal neuralgia. Front Neuroanat. 2016;10:95.

7. Katusic S, Beard CM, Bergstralh E, Kurland LT. Incidence and clinical features of trigeminal neuralgia, Rochester, Minnesota, 1945-1984. Ann Neurol. 1990;27:89-95.

8. Gronseth G, Cruccu G, Alksne J, et al. Practice parameter: the diagnostic evaluation and treatment of trigeminal neuralgia (an evidence-based review): report of the Quality Standards Subcommittee of the American Academy of Neurology and the European Federation of Neurological Societies. Neurology. 2008;71:1183-1190.

9. Campbell FG, Graham JG, Zilkha KJ. Clinical trial of carbazepine (tegretol) in trigeminal neuralgia. J Neurol Neurosurg Psychiatry. 1966;29:265-267.

10. Fromm GH, Terrence CF, Chattha AS. Baclofen in the treatment of trigeminal neuralgia: double-blind study and long-term follow-up. Ann Neurol. 1984;15:240-244.

11. Zakrzewska JM, Lopez BC, Kim SE, Coakham HB. Patient reports of satisfaction after microvascular depression and partial sensory rhizotomy for trigeminal neuralgia. Neurosurgery. 2005;56:1304-1311.

12. A Nanda, V Javalkar, S Zhang, O Ahmed. Long term efficacy and patient satisfaction of microvascular decompression and gamma knife radiosurgery for trigeminal neuralgia. Journal of Clinical Neuroscience 2015;22:818-822.

13. Linskey ME, Ratanatharathorn V, Peñagaricano J. A prospective cohort study of microvascular decompression and Gamma Knife surgery in patients with trigeminal. J Neurosurg. 2008;109 Suppl:160-172.

14. Zakrzewska JM, Thomas DG. Patient's assessment of outcome after three surgical procedures for the management of trigeminal neuralgia. Acta Neurochir (Wien). 1993;122:225-230.

15. Sindrup SH, Jensen TS.. Pharmacotherapy of trigeminal neuralgia. Clin J Pain 2002;18:22-27.

16. Wiffen PJ, McQuay HJ, Moore RA. Carbamazepine for acute and chronic pain. Cochrane

For peer review only - http://bmjopen.bmj.com/site/about/guidelines.xhtml

Database Syst Rev. 2005;(3):CD005451.

17. Zakrzewska JM, Patsalos PN. Long-term cohort study comparing medical (oxcarbazepine) and surgical management of intractable trigeminal neuralgia. Pain. 2002;95:259-66

18. Zakrzewska JM, Chaudhry Z, Nurmikko TJ, et al. Lamotrigine (Lamictal) in refractory trigeminal neuralgia: results from a double-blind placebo controlled crossover trial. Pain. 1997;73:223-230.

19. Court JE, Kase CS. Treatment of tic douloureux with a new anticonvulsant (clonazepam). J Neurol Neurosurg Psychiatry. 1976;39:297-299.

20. Yuan M, Zhou HY, Xiao ZL, et al. Efficacy and Safety of Gabapentin vs. Carbamazepine in the Treatment of Trigeminal Neuralgia: A Meta-Analysis. Pain Pract. 2016;16:1083-1091

21. Obermann M, Yoon MS, Sensen K, Maschke M, Diener HC, Katsarava Z. Efficacy of pregabalin in the treatment of trigeminal neuralgia. Cephalalgia. 2008;28:174-181.

22. Peiris JB, Perera GL, Devendra SV, Lionel ND. Sodium valproate in trigeminal neuralgia. Med J Aust. 1980;2:278.

23. Zakrzewska JM, Linskey ME. Trigeminal neuralgia. Clin Evid. 2014;10:1207

24. McQuay H, Carroll D, Jadad AR, et al. Anticonvulsant drugs for management of pain: a systematic review. BMJ. 1995;311:1047-1052.

25. Wiffen PJ, Rees J. Lamotrigine for acute and chronic pain. Cochrane Database Syst Rev. 2007;(2):CD006044.

26. Wiffen PJ, McQuay HJ, Rees J, Moore RA. Gabapentin for acute and chronic pain. Cochrane Database Syst Rev. 2005;(3):CD005452.

27. Wiffen PJ, Derry S, Moore RA, McQuay HJ. Carbamazepine for acute and chronic pain in adults. Cochrane Database Syst Rev. 2011;(1):CD005451.

28. Moore RA, Straube S, Wiffen PJ, Derry S, McQuay HJ. Pregabalin for acute and chronic pain in adults. Cochrane Database Syst Rev. 2009;(3):CD007076.

29. Caldwell DM, Ades AE, Higgins JP. Simultaneous comparison of multiple treatments: combining direct and indirect evidence. BMJ. 2005;331:897-900.

30. Cipriani A, Higgins JP, Geddes JR, Salanti G. Conceptual and technical challenges in network meta-analysis. Ann Intern Med. 2013;159:130-137.

31. Cipriani A, Furukawa TA, Salanti G, et al. Comparative efficacy and acceptability of 12 new-generation antidepressants: a multiple-treatments meta-analysis. Lancet. 2009;373:746-758.

32. Shamseer L, Moher D, Clarke M, et al. Preferred reporting items for systematic review and meta-analysis protocols (PRISMA-P) 2015: elaboration and explanation. BMJ. 2015;349:g7647.

33. Hutton B, Salanti G, Caldwell DM, et al. The PRISMA Extension Statement for Reporting of Systematic Reviews Incorporating Network Meta-analyses of Health Care Interventions: Checklist and Explanations. Ann Intern Med. 2015;162:777-784.

34. Merskey H, Bogduk N. Classification of chronic pain. Descriptions of chronic pain syndromes and definitions of pain terms, IASP Press, Seattle 1994, pp. 59-71.

For peer review only - http://bmjopen.bmj.com/site/about/guidelines.xhtml

For peer review only

35. Zakrzewska JM. Diagnosis and differential diagnosis of trigeminal neuralgia. Clin J Pain. 2002;18:14-21.

36. Dworkin RH, Turk DC, Farrar JT, et al. Core outcome measures for chronic pain clinical trials: IMMPACT recommendations. Pain. 2005;113:9-19.

37. Ware JE Jr, Sherbourne CD. The MOS 36-item short-form health survey (SF-36). I. Conceptual framework and item selection. Med Care. 1992;30:473-483.

38. Qin Z, Li B, Wu J, et al. Acupuncture for chronic diarrhea in adults: protocol for a systematic review. Medicine (Baltimore). 2017;94:e5952.

39. Higgins JPT, Altman DG, Sterne JAC (editors). Chapter 8: Assessing risk of bias in included studies. In: Higgins JPT, Green S(editors). Cochrane Handbook for Systematic Reviews of Intervention Version 5.1.0 [updated March 2011]. The Cochrane Collaboration, 2011. Available from www.cochrane-handbook.org.

40. Salanti G, Del Giovane C, Chaimani A, et al. Evaluating the quality of evidence from a network meta-analysis. PLoS ONE. 2014;9:e99682.

41. Furukawa TA, Cipriani A, Barbui C, Brambilla P, Watanabe N. Imputing response rates from means and standard deviations in meta-analyses. Int Clin Psychopharmacol. 2005;20:49-52.

42. Lin L, Zhang J, Hodges J, Chu H. Performing arm-based network meta-analysis in R with the pcnetmeta package. Journal of Statistical Software. 2017;doi: 10.18637/jss.v080.i05

43. Lin L, Zhang J, Chu H. pcnetmeta: Methods for patient-centered network meta-analysis. 2016. R package version 2.4 http://CRAN.R-project.org/package=pcnetmeta

44. Zhang J, Carlin B.P, Neaton J.D, et al. Network meta-analysis of randomized clinical trials: Reporting the proper summaries. Clinical Trials. 2014;11:246-262

45. Higgins JP, Thompson SG, Deeks JJ, Altman DG. Measuring inconsistency in meta-analyses. BMJ. 2003;327:557-560.

46. Baker SG, Kramer BS. The transitive fallacy for randomized trials: if A bests B and B bests C in separate trials, is A better than C? BMC Med Res Methodol. 2002;2:13.

47. White IR, Barrett JK, Jackson D, et al. Consistency and inconsistency in network meta-analysis: model estimation using multivariate meta-regression. Res Synth Methods. 2012;3:111-125

48. Higgins JPT, Jackson D, Barrett JL, et al. Consistency and inconsistency in network meta-analysis: concepts and models for multi-arm studies. Res Synth Methods. 2012;3:98-110

49. Dias S, Welton NJ, Caldwell DM, et al. Checking consistency in mixed treatment comparison meta-analysis. Stat Med. 2010;29:932-944

50. Lu G, Ades AE. Assessing evidence inconsistency in mixed treatment comparisions. J Am Stat Assoc. 2006;101:447-459.

51. Veroniki AA, Vasiliadis HS, Higgins JP, Salanti G. Evaluation of inconsistency in networks of interventions. Int J Epidemiol. 2013;42:332-345.

For peer review only - http://bmjopen.bmj.com/site/about/guidelines.xhtml

Figure legends of figure:

Figure 1. Network plot of all possible direct comparisons between the eligible interventions.

Figure 2. PRISMA flow chart.

For peer review only

For peer review only - http://bmjopen.bmj.com/site/about/guidelines.xhtml

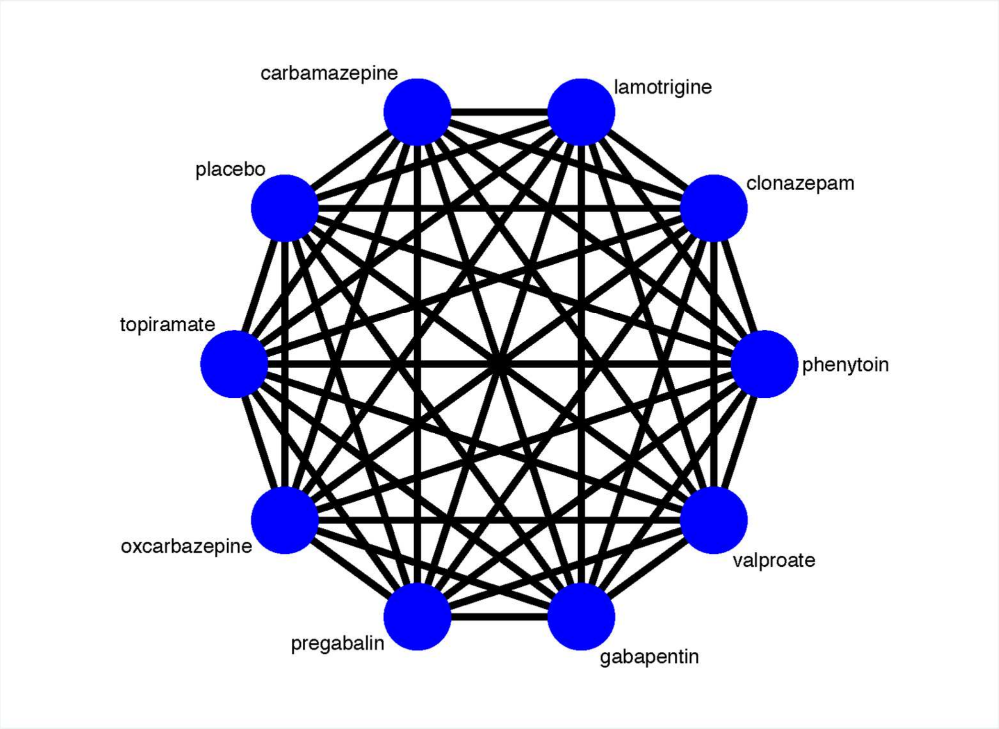

Network plot of all possible direct comparisons between the eligible interventions.

140x101mm (300 x 300 DPI)

For peer review only

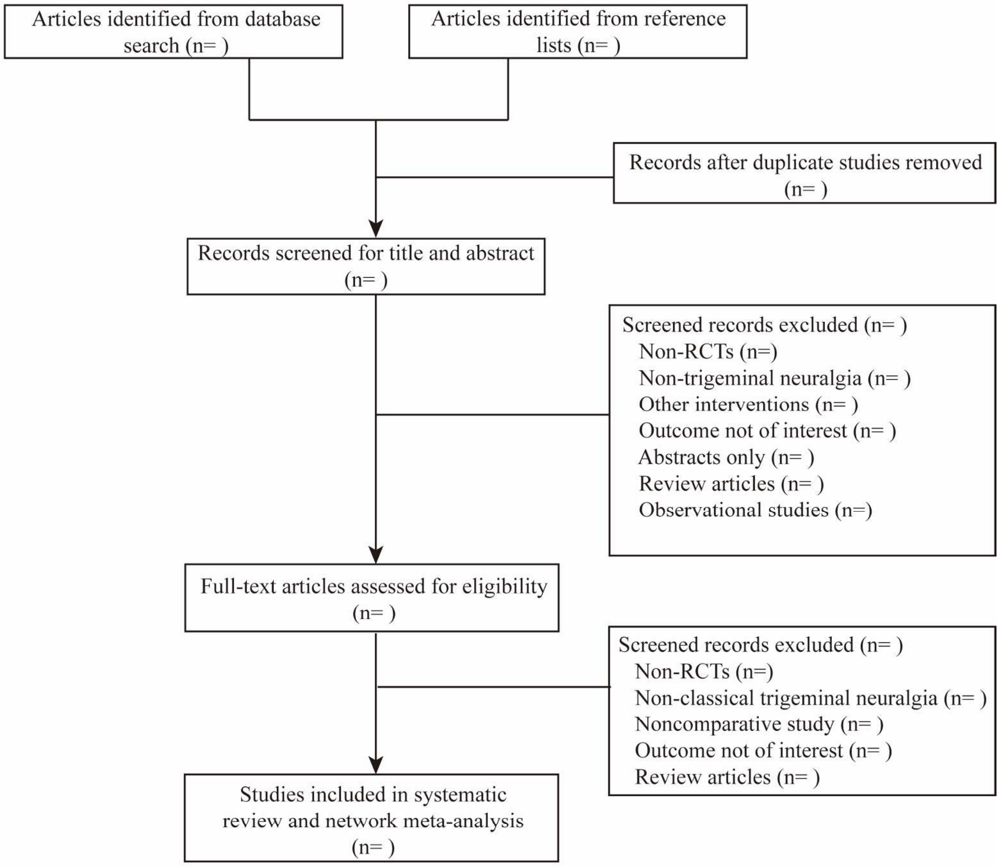

PRISMA flow chart

192x219mm (300 x 300 DPI)

PRISMA-P (Preferred Reporting Items for Systematic review and Meta-Analysis Protocols) 2015 checklist: recommended items to address in a systematic review protocol*

Section and topic	Item No	Checklist item
**ADMINISTRATIVE INFORMATION**		
Title:		
Identification	1a	Identify the report as a protocol of a systematic review Page 1
Update	1b	If the protocol is for an update of a previous systematic review, identify as such NA
Registration	2	If registered, provide the name of the registry (such as PROSPERO) and registration number Page 2
Authors:		
Contact	3a	Provide name, institutional affiliation, e-mail address of all protocol authors; provide physical mailing address of corresponding author Page 1
Contributions	3b	Describe contributions of protocol authors and identify the guarantor of the review Page 11
Amendments	4	If the protocol represents an amendment of a previously completed or published protocol, identify as such and list changes; otherwise, state plan for documenting important protocol amendments Page 11
Support:		
Sources	5a	Indicate sources of financial or other support for the review Page 11
Sponsor	5b	Provide name for the review funder and/or sponsor Page 11
Role of sponsor or funder	5c	Describe roles of funder(s), sponsor(s), and/or institution(s), if any, in developing the protocol Page 11
**INTRODUCTION**		
Rationale	6	Describe the rationale for the review in the context of what is already known Page 3
Objectives	7	Provide an explicit statement of the question(s) the review will address with reference to participants, interventions, comparators, and outcomes (PICO) Page 4
**METHODS**		
Eligibility criteria	8	Specify the study characteristics (such as PICO, study design, setting, time frame) and report characteristics (such as years considered, language, publication status) to be used as criteria for eligibility for the review Page 4
Information sources	9	Describe all intended information sources (such as electronic databases, contact with study authors, trial registers or other grey literature sources) with planned dates of coverage Page 5
Search strategy	10	Present draft of search strategy to be used for at least one electronic database, including planned limits, such that it could be repeated Page 6
Study records:		
Data management	11a	Describe the mechanism(s) that will be used to manage records and data throughout the review Page 6-7

For peer review only

Selection process	11b	State the process that will be used for selecting studies (such as two independent reviewers) through each phase of the review (that is, screening, eligibility and inclusion in meta-analysis) Page 6-7
Data collection process	11c	Describe planned method of extracting data from reports (such as piloting forms, done independently, in duplicate), any processes for obtaining and confirming data from investigators Page 6-7
Data items	12	List and define all variables for which data will be sought (such as PICO items, funding sources), any pre-planned data assumptions and simplifications Page 6-7
Outcomes and prioritization	13	List and define all outcomes for which data will be sought, including prioritization of main and additional outcomes, with rationale Page 5-6
Risk of bias in individual studies	14	Describe anticipated methods for assessing risk of bias of individual studies, including whether this will be done at the outcome or study level, or both; state how this information will be used in data synthesis Page 7
Data synthesis	15a	Describe criteria under which study data will be quantitatively synthesised Page 7-8
	15b	If data are appropriate for quantitative synthesis, describe planned summary measures, methods of handling data and methods of combining data from studies, including any planned exploration of consistency (such as I^2, Kendall's τ) Page 8
	15c	Describe any proposed additional analyses (such as sensitivity or subgroup analyses, meta-regression) Page 9-10
	15d	If quantitative synthesis is not appropriate, describe the type of summary planned Page 9-10
Meta-bias(es)	16	Specify any planned assessment of meta-bias(es) (such as publication bias across studies, selective reporting within studies) Page 7
Confidence in cumulative evidence	17	Describe how the strength of the body of evidence will be assessed (such as GRADE) Page 7

*** It is strongly recommended that this checklist be read in conjunction with the PRISMA-P Explanation and Elaboration (cite when available) for important clarification on the items. Amendments to a review protocol should be tracked and dated. The copyright for PRISMA-P (including checklist) is held by the PRISMA-P Group and is distributed under a Creative Commons Attribution Licence 4.0.**

From: Shamseer L, Moher D, Clarke M, Ghersi D, Liberati A, Petticrew M, Shekelle P, Stewart L, PRISMA-P Group. Preferred reporting items for systematic review and meta-analysis protocols (PRISMA-P) 2015: elaboration and explanation. BMJ. 2015 Jan 2;349(jan02 1):g7647.

For peer review only

PubMed

Patient

#1 "Trigeminal Neuralgia"[Mesh]

#2 Trigeminal Neuralgia*[Title/Abstract] OR Trifacial Neuralgia*[Title/Abstract] OR Fothergill Disease[Title/Abstract] OR Tic Douloureux[Title/Abstract] OR Epileptiform Neuralgia*[Title/Abstract] OR trigeminus neuralgia[Title/Abstract] OR prosopalgia[Title/Abstract] OR prosoponeuralgia[Title/Abstract] OR trigeminal nerve neuralgia[Title/Abstract] OR trigeminal nerve neuropathy[Title/Abstract] OR trigeminal neuropathy[Title/Abstract] OR trigeminus nerve neuralgia[Title/Abstract] OR trigeminus nerve neuropathy[Title/Abstract]

#3 #1 OR #2

RCT

#4 "Clinical Trials, Phase II as Topic"[Mesh] OR "Clinical Trials, Phase III as Topic"[Mesh] OR "Clinical Trials, Phase IV as Topic"[Mesh] OR "Controlled Clinical Trials as Topic"[Mesh] OR "Randomized Controlled Trials as Topic"[Mesh] OR "Intention to Treat Analysis"[Mesh] OR "Pragmatic Clinical Trials as Topic"[Mesh] OR "Clinical Trials, Phase II"[Publication Type] OR "Clinical Trials, Phase III"[Publication Type] OR "Clinical Trials, Phase IV"[Publication Type] OR "Controlled Clinical Trials"[Publication Type] OR "Randomized Controlled Trials"[Publication Type] OR "Pragmatic Clinical Trials as Topic"[Publication Type] OR "Single-Blind Method"[Mesh] OR "Double-Blind Method"[Mesh]

#5 random*[Title/Abstract] OR blind*[Title/Abstract] OR singleblind*[Title/Abstract] OR doubleblind*[Title/Abstract] OR trebleblind*[Title/Abstract] OR tripleblind*[Title/Abstract]

#6 #4 OR #5

#7 #3 AND #6

EMBASE.com

Patient

#1 'trigeminus neuralgia'/exp

#2 'trigeminal neuralgias':ab,ti OR 'trifacial neuralgias':ab,ti OR 'trigeminal neuralgia':ab,ti OR 'trifacial neuralgia':ab,ti OR 'fothergill disease':ab,ti OR 'tic douloureux':ab,ti OR 'epileptiform neuralgia':ab,ti OR 'epileptiform neuralgias':ab,ti OR 'trigeminus neuralgia':ab,ti OR prosopalgia:ab,ti OR prosoponeuralgia:ab,ti OR 'trigeminal nerve neuralgia':ab,ti OR 'trigeminal nerve neuropathy':ab,ti OR 'rigeminal neuropathy':ab,ti OR 'trigeminus nerve neuralgia':ab,ti OR 'trigeminus nerve neuropathy':ab,ti

#3 #1 OR #2

RCT

#4 'multicenter study (topic)'/exp OR 'phase 2 clinical trial (topic)'/exp OR 'phase 3 clinical trial

For peer review only

(topic)'/exp OR 'phase 4 clinical trial (topic)'/exp OR 'controlled clinical trial (topic)'/exp OR 'randomized controlled trial (topic)'/exp OR 'single blind procedure'/exp OR 'double blind procedure'/exp

#5 random*:ab,ti OR blind*:ab,ti OR singleblind*:ab,ti OR doubleblind*:ab,ti OR trebleblind*:ab,ti OR tripleblind*:ab,ti

#6 #4 OR #5

#7 #3 AND #6

Cochrane Library

#1 MeSH descriptor: [Trigeminal Neuralgia] explode all trees

#2 Trigeminal Neuralgia*:ti,ab,kw or Trifacial Neuralgia*:ti,ab,kw or Fothergill Disease:ti,ab,kw or Tic Douloureux:ti,ab,kw or Epileptiform Neuralgia*:ti,ab,kw or trigeminus neuralgia:ti,ab,kw or prosopalgia:ti,ab,kw or prosoponeuralgia:ti,ab,kw or trigeminal nerve neuralgia:ti,ab,kw or trigeminal nerve neuropathy:ti,ab,kw or trigeminal neuropathy:ti,ab,kw or trigeminus nerve neuralgia:ti,ab,kw or trigeminus nerve neuropathy:ti,ab,kw

#3 #1 or #2

For peer review only
